# HHO5 orchestrates dose-dependent feedback regulation of organic versus inorganic nitrogen signaling in Arabidopsis

**DOI:** 10.1093/plcell/koag201

**Published:** 2026-07-30

**Authors:** Will E Hinckley, Joseph Swift, Francisco Romei, Jorge P Muschietti, Samantha Frangos, Shao-shan Carol Huang, Gloria M Coruzzi, Mariana Obertello

**Affiliations:** Center for Genomics and Systems Biology, Department of Biology, New York University, 12 Waverly Pl, New York, NY 10003, United States; Center for Genomics and Systems Biology, Department of Biology, New York University, 12 Waverly Pl, New York, NY 10003, United States; Instituto de Investigaciones en Ingeniería Genética y Biología Molecular, Dr. Héctor Torres (INGEBI-CONICET), Vuelta de Obligado 2490, Buenos Aires C1428ADN, Argentina; Departamento de Fisiología y Biología Molecular, Facultad de Ciencias Exactas y Naturales, Universidad de Buenos Aires, Int. Güiraldes 2160, Ciudad Universitaria, Pabellón II, Buenos Aires C1428EGA, Argentina; Instituto de Investigaciones en Ingeniería Genética y Biología Molecular, Dr. Héctor Torres (INGEBI-CONICET), Vuelta de Obligado 2490, Buenos Aires C1428ADN, Argentina; Departamento de Biodiversidad y Biología Experimental, Facultad de Ciencias Exactas y Naturales, Universidad de Buenos Aires, Int. Güiraldes 2160, Ciudad Universitaria, Pabellón II, Buenos Aires C1428EGA, Argentina; Center for Genomics and Systems Biology, Department of Biology, New York University, 12 Waverly Pl, New York, NY 10003, United States; Center for Genomics and Systems Biology, Department of Biology, New York University, 12 Waverly Pl, New York, NY 10003, United States; Center for Genomics and Systems Biology, Department of Biology, New York University, 12 Waverly Pl, New York, NY 10003, United States; Instituto de Investigaciones en Ingeniería Genética y Biología Molecular, Dr. Héctor Torres (INGEBI-CONICET), Vuelta de Obligado 2490, Buenos Aires C1428ADN, Argentina; Departamento de Fisiología y Biología Molecular, Facultad de Ciencias Exactas y Naturales, Universidad de Buenos Aires, Int. Güiraldes 2160, Ciudad Universitaria, Pabellón II, Buenos Aires C1428EGA, Argentina

## Abstract

A major goal in agriculture is to engineer crops to maintain yield with less nitrogen (N) fertilizer. Important regulators of plant N-responses include HRS1 HOMOLOG (HHO) transcription factors (TFs); yet, their redundant repressive mode of action hinders functional analyses. Here, we highlight HHO5 as a unique HHO TF based on its phylogenetic position, phloem specific expression, and dual role in regulating responses to inorganic and organic N-dose signals. Our results support a model whereby HHO5 mediates feedback repression of inorganic nitrate uptake under organic N satiety: (i) Meta-analyses revealed *HHO5* expression is repressed by inorganic N, but induced by organic N treatments. (ii) HHO5 directly binds and represses nitrate response genes, but indirectly induces organic N-response genes. (iii) HHO5 indirect target gene induction occurs via WRKY partner TFs, validated using a cell-based DoubleTARGET TF co-perturbation assay. (iv) HHO5 initiates a validated gene regulatory network path which encompasses ∼12% of the N-dose response genes *in planta*. (v) Phenotypically, single *hho5* T-DNA mutants show reduced N-dose dependent growth, and decreased seed N content. (vi) HHO5 represses nitrate uptake but induces root growth responses to Glu *in planta*. Overall, our findings support a model wherein HHO5 orchestrates dose dependent feedback regulation of organic versus inorganic N-signaling in Arabidopsis.

## Introduction

Nitrogen (N) is an essential nutrient for plant growth. Biomass, grain yield, root architecture, and photosynthesis are all impacted by N-availability. However, plants only absorb about 50% of available N from fertilizers, which are costly and harm the environment (Raun et al. 1999). For these reasons, the demand for crops that can maintain yield with less N-fertilizer is rising (Li et al. 2017). In order to achieve this, we must first understand how plants control N uptake and assimilation, and how these signals can be manipulated to optimize plant growth in low N environments.

N-adaptive responses are initiated at the molecular level, where environmental stimuli trigger signaling cascades that rewire gene networks, which in turn alters plant growth and development. One such sensor of N signals is *NRT1.1*, which acts as an N-signaling transceptor (transporter and sensor, Bouguyon et al. 2015). Additional mechanisms are being uncovered, for example, the transcription factor (TF) *NLP7* was shown to act as a N sensor by directly binding nitrate (Liu et al. 2022b). While individual low and high affinity N sensors have been previously characterized (Okamoto et al. 2006; Yuan et al. 2007; Ho et al. 2009), how plants relay quantitative N-signals across N-doses at the transcriptome level has received less attention.

N-signals are highly dynamic, varying across the dimensions of both dose and time (Krouk et al. 2010b; Swift et al. 2020; Shanks et al. 2024). Previous work that integrated N-dose-by-time treatments of Arabidopsis seedlings revealed that temporal responses to N-dose signals can be modeled by simple Michaelis-Menten (MM) kinetics (Swift et al. 2020). Because TFs establish the rates by which transcription takes place, they can be conceptually compared to catalytic enzymes in a MM model (Swift et al. 2020), although this analogy is not strict (Noviello 2025). In support of the MM model, overexpression of the TF *TGA1* increased the Vmax of N-dose responsive mRNAs, and accelerated N-dependent plant growth rates (Swift et al. 2020). Perturbation of *TGA1* explained misregulation of ∼10% of the MM N-dose responsive genes *in planta*, suggesting that other transcriptional regulators of N-dose signaling are yet to be found.

Another advantage of studying N-dose response genes over time is the ability to uncover early acting transcriptional regulators. The first TFs whose expression levels respond to N-stimuli (within minutes) have been shown to play key roles in regulating downstream genes in N-related pathways (Krouk et al. 2010b; Varala et al. 2018; Brooks et al. 2019; Swift et al. 2020; Shanks et al. 2024). TFs that respond early to N signals include *CRF4,* as well as several members of the *HRS1-HOMOLOG* (*HHO*) TF family (*HHO2, HHO3,* and *HHO5*) (Varala et al. 2018).

The *HHO* TFs are a sub-family of the G2-like class of plant MYB TFs, some of which are known for their role in repressing N responses (Li et al. 2021). Of the 7 HHO genes, 4 members (*HRS1, HHO1*, *HHO2, HHO3*) form a group of closely related TFs called the *NITRATE-INDUCIBLE, GARP-TYPE TRANSCRIPTIONAL REPRESSOR1* proteins (*NIGT1s*). *NIGT1* TFs redundantly repress high-affinity nitrate transporters (Kiba et al. 2018). *NIGT1* TFs more strongly influence N-related phenotypes when mutated in higher-order combinations due to their redundancy (Safi et al. 2021). Thus, N-signal repression by NIGT1 TFs may be agriculturally important, but their redundancy makes them difficult targets for gene editing applications.

In contrast, *HHO4*, *HHO5*, and *HHO6* are more phylogenetically diverged from the *NIGT1* TFs (Li et al. 2021; Safi et al. 2021), and their role in N-signaling has not been well studied. Notably, *HHO5* was previously shown to act as a repressor of genes expressed in the floral meristem (Moreau et al. 2016). In previous studies, *HHO5* emerged as one of the earliest responders to fine time-scale N-treatments (Krouk et al. 2010b; Varala et al. 2018). Swift et al. 2020 showed that the gene expression of *HHO5* in response to N-dose follows MM kinetics and is regulated by the master TF *TGA1* (Swift et al. 2020). Moreover, *HHO5* was previously identified to be evolutionarily conserved in an N-response network shared between Arabidopsis and rice (Obertello et al. 2015). *HHO5* is thus a strong candidate regulator of plant N-dose signaling.

In this study, we characterize the role of HHO5 in orchestrating feedback regulation between organic and inorganic N-dose dependent signaling in Arabidopsis. We show that *HHO5* regulates the amplitude of N-dose response for hundreds of genes *in planta* and is specifically expressed in phloem, a conduit for N-transport. Unlike the NIGT1 TFs, HHO5 plays a non-redundant role in vivo, as single *hho5* T-DNA mutants dampen plant growth in an N-dose dependent manner. While HHO5 directly represses inorganic N-response genes, it also indirectly activates many organic-N response genes, contrasting with the known repressive behavior of the NIGT1 TFs. We propose and validate a model where indirect activation of gene expression by HHO5 is mediated by partner TFs, such as the putative partner WRKY21. We constructed a gene regulatory network downstream of HHO5 that encompasses both inorganic and organic N signals. This experimentally validated network path can account for the regulation of ∼70% of the HHO5-targeted N-dose genes *in planta*. In total, our GRN of HHO5 direct and indirect targets encompasses 12% of the total N-dose response genes *in planta*.

To better clarify the dual inducing/repressing role of HHO5 over N-dose signaling, we completed a meta-analysis of N-related genomic datasets. The meta-analysis revealed that *HHO5* expression is repressed by nitrate signals but induced by organic N signals. Based on these results, we constructed a model in which *HHO5* responds to organic N to initiate repression of nitrate uptake via *NRT1.1*. In addition, in response to organic N stimulus, HHO5 induces the expression of glutamate receptors related to organic N signaling and defense (Grenzi et al. 2022; Lee et al. 2023), and hundreds of genes that respond to exogenous Glu-treatments (Goto et al. 2020) (Graphical Abstract). Together, our model proposes that HHO5 orchestrates feedback repression from organic N and defense signals onto nitrate uptake and transport as a means of conserving energy. To validate this model, our physiology experiments show the *hho5-2* mutant displays increased inorganic N uptake at low N doses and dose-dependent deficits in primary root responses to organic-N (Glu). Overall, we propose a dual regulatory role of the TF HHO5 in balancing inorganic vs organic N-dose signaling in Arabidopsis.

## Materials and methods

### Arabidopsis *hho5* mutants

Two Arabidopsis T-DNA mutants in the *HHO5* (AT4G37180) gene obtained from ABRC included *hho5-*1 (SAIL_806_F06) and *hho5*-2 (SALK_077802) hosting T-DNA insertions in the first and fifth exons, respectively (O’Malley and Ecker 2010). Homozygous lines of each allele were obtained and their genotypes confirmed by PCR after 2 backcrosses to the parental Col-0 ecotype to remove extra T-DNA insertions. Left border sequencing of the T-DNA was conducted to verify the insertion site of each allele. To test for *hho5* transcript, total RNA was extracted from 2-wk-old wild-type and mutant plants grown on MS media (Murashige and Skoog 1962) and reverse transcribed. Both, *hho5*-1 and *hho5*-2 showed the absence of *hho5* transcript in seedlings when using primers designed to amplify 3´UTR transcript.

### Mutant phenotype analysis of *hho5*

Seeds were sown on vertical 120-by-120 mm square plates containing 50 mL of MS basal salts, supplemented with 3 mM sucrose, and 0.8% (w/v) agar at pH 5.7 and KNO_3_ or/and NH_4_NO_3_. For all root growth, 8 to 10 plants spread across 3 separate plate replicates were grown under long-day conditions (16 h light, 8 h dark), with a fluorescent light intensity of 120 μmol m^−2^s^−1^ at constant temperature of 22 °C. For the L-Glutamate (Glu) and L- Glutamine (Gln) assays, 4-d-old plants grew on 1 mM KNO_3_ were transferred to vertical plates with Glu or Gln as organic N organic sources at different concentrations (5, 10, 15, or 20 mM). In all cases, surface-sterilized seeds were aligned on the plate surface; mutant lines and wild-type Col-0 were placed on the same plate. Primary root length was scored every 3 d. The root length was quantified using ImageJ 1.52a software (National Institutes of Health). At the end of the experiment, plant shoots were extracted, and fresh and dry weight were measured. For the measurement of dry weight, tissue was dried at 70 °C for 3 d and weighed to determine dry weight. Silique length was evaluated in mature Col-0 and *hho5* mutant plants. 10 plants of each line were used for silique and seed counting (3 siliques per plant). Siliques from the middle part of the main inflorescence were analyzed. Seed number per silique was recorded for each line. All data are expressed as means ± SEM. We found that phenotypic responses were the same regardless whether the N source was KNO_3_ or NH_4_NO_3_. Influx of 15N was assayed in plants grown for 14 d as described above, on either 1 or 10 mM supplied as K^15^NO_3_ or ^15^NH_4_^15^NO_3_. Immediately after treatment, plants were rinsed in 10 mM CaSO_4_, roots and shoots were separated. Six plants per condition were studied. Tissues were dried at 70 °C for 48 h prior to analysis. The abundance of ^15^N in NH_4_ and NO_3_ was measured by mass spectrometry at the UC Davis Stable Isotope Facility. Comparisons among groups were shown using ANOVA with Tukey post hoc tests. Total N content in dried seeds was determined by the Kjeldahl method.

### Histochemical staining and image analysis

p*1kbHHO5::GUS* seedlings were grown for 10 d on MS media, supplemented with 1 mM KNO_3_ and 1% sucrose. After 10 d, plants were starved of N for an additional day. Two hours after subjective dawn on the 12th day, plants were treated with no N, 5 mM or 10 mM KNO_3_ for 2 h. After these 2 h treatments, 2 to 3 plants per 3 independent experiments were collected and the histochemical analysis of GUS reporter enzyme activity was performed as described by Weigel 2002. The staining patterns of GUS in the roots were visualized under the Olympus BX51 microscope (Olympus, Tokyo, Japan) with differential interference contrast optics.

### N-dose treatments of WT and *hho5* mutants

We grew ∼100 seedlings Col-0, *hho5-1* and *hho5-2* mutants of *Arabidopsis* in hydroponics. Seedlings were grown for 13 d on MS media, supplemented with 1 mM KNO_3_ and 1% sucrose. Plants were grown under long-day conditions (16 h light, 8 h dark), with a light intensity of 120 μmol m^−2^s^−1^ at constant temperature of 22 °C in liquid media (phytatrays). After 13 d, plants were starved of N for an additional day. Two hours after subjective dawn on the 15th day, plants were transferred to hydroponic treatments of one of 6 N doses and left suspended for 2 h. All treatments were performed on nutrient media identical in composition except for N concentration or KCl (control). N was supplied as a mixture of KNO_3_ and NH_4_NO_3_ at final total N concentrations of 0, 0.006, 0.06, 0.6, 6, and 60 mM. Additionally, 1 set of plants were treated with 20 mM KCl to control for the effect of potassium. Plants were treated in triplicate (treatment replicates, 3 different phytatray plant transfers per treatment), resulting in 63 samples total. After a 2-h incubation period, root tissue was excised and flash frozen in liquid nitrogen. This experimental design is consistent with our previous studies optimized to capture early responses to N treatment (Varala et al. 2018; Swift et al. 2020). RNA was extracted from whole roots using the Qiagen RNeasy kit with on-column DNAse digestion and quality checked using Agilent Tapestation HSRNA Kit. mRNA was isolated using oligo-dT purification from 1 μg of total RNA (Thermo Fisher Scientific) and libraries made using NEBNext RNA Kit. Libraries were sequenced using Illumina NextSeq with 1 × 75 bp read chemistry. Resulting reads were aligned to the TAIR10 genome using Tophat (Trapnell et al. 2009) and aligned to the Araport11 *Arabidopsis* annotation (Cheng et al. 2017) using HT-Seq (Anders et al. 2015) to call gene counts (Raw Gene Expression can be found in Table S10).

### N-dose DEGs: calling and visualizing with DESeq2

Raw gene counts were used in DESeq2 (Love et al. 2014) for normalization. VST normalized counts of the top 5000 most variable genes were put into the DESeq2 PlotPCA function to visualize the entire transcriptome dataset. N-dose DEGs were called using the DESeq2 LRT Test full model ∼ Genotype + Dose + Treatment, where genotype referred to WT, *hho5-1*, and *hho5-2* mutants. Dose referred to the numerical dose of nutrient treated. The Treatment term differentiated the N treatment from the KCl treatment control. 0 mM was classified as part of the control treatment. The LRT test reduced model ∼ Genotype + Treatment was used to call 4,644 N-dose DEGs (*P-*adj <0.01). DEGs were clustered and visualized using the DEGReport package in R using consensusCluster set to True and minimum cluster (minc) set to 10 genes (Pantano 2025). Gene Ontology enrichment analysis was completed on these clusters using the clusterProfiler package in R, with ontology set to “BP,” FDR and *P*-value set to 0.05, and KetType set to TAIR (Yu et al. 2012). Additional clusters were identified using DEGReport with consensusCluster set to False. N metabolism genes were identified using the KEGG database (Kanehisa et al. 2025). These N-metabolism genes were intersected with the 4,464 N-dose genes, and scaled gene expression for the N-dose responsive N-metabolism genes was visualized in a heatmap using the ComplexHeatmap package in R (Gu et al. 2016). The expression of individual genes was plotted using the DESeq2 PlotCounts function and ggplot2 (Wickham 2016).

### Analysis of HHO TF family N-dose response

Gene expression of the HHO TFs was plotted across N-doses tested using ggplot2. Protein sequences of the 7 HHO TFs were acquired from TAIR.org and used as input for MEGA7. The evolutionary history was inferred by using the Maximum Likelihood method based on the w/freq. model (Jones et al. 1992). The bootstrap consensus tree inferred from 1,000 replicates is taken to represent the evolutionary history of the taxa analyzed (Felsenstein 1985). Branches corresponding to partitions reproduced in less than 50% bootstrap replicates are collapsed. The percentage of replicate trees in which the associated taxa clustered together in the bootstrap test (1,000 replicates) are shown next to the branches (Felsenstein 1985). Initial tree(s) for the heuristic search were obtained by applying the Neighbor-Joining method to a matrix of pairwise distances estimated using a JTT model. The analysis involved 7 amino acid sequences. All positions containing gaps and missing data were eliminated. There were a total of 273 positions in the final dataset. Evolutionary analyses were conducted in MEGA7 (Kumar et al. 2016). Cell-type expression profiles were generated by plotting a heatmap of the average gene expression of the HHO TFs across cell types from the Arabidopsis root cell type atlas from Li et al. 2016.

### Identification of HHO5-regulated N-dose response genes in planta with DESeq2

**Figure 1:**
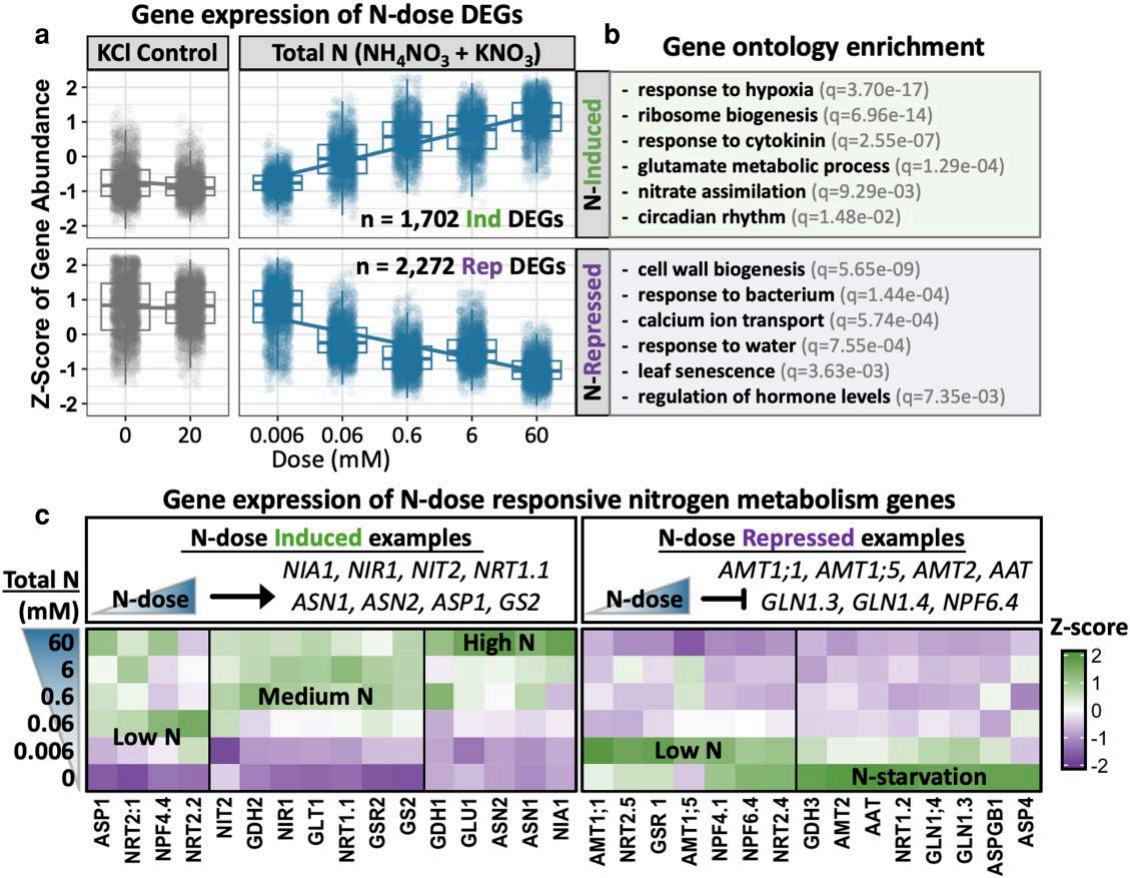
N-dose responses reprogram the root transcriptome of Arabidopsis. a) N-dose response genes induced (top) or repressed (bottom) relative to KCl treatment controls (Z-score scaled expression), from a total of 4,464 N-dose DEGs (DESeq2 *P*.adj < 0.05). Total N-dose in mM = 1xNH_4_ (NH_4_NO_3_) + 2xNO_3_ (KNO_3_ and NH_4_NO_3_) (eg Total N-dose of 60 mM = 20 mM NH_4_NO_3_ and 20 mM KNO_3_). b) Top-ranked non-redundant enriched GO terms in the N-dose induced or repressed gene clusters, q-value of enrichment significance. c) Significant N-dose responses of genes involved in N-uptake/reduction/assimilation ordered by hierarchical clustering of Z-score scaled expression.

Using the same DESeq2 object as before, HHO5 genotype DEGs were called using the standard DESeq2 wald test for each allele separately. This was accomplished by setting contrasts for (*hho5-1* vs WT) and (*hho5-2* vs WT) with *P-*adj <0.05. We then took the intersection of the *hho5-1* and *hho5-2* DEGs, resulting in 2,894 high confidence DEGs called in both mutant alleles. The significant overlap between these 2 lists was tested using a hypergeometric test in R (phyper). These 2,894 HHO5-regulated genes were intersected with the N-dose genes identified in WT, and the 828 overlapping genes represented a significant intersection based on a hypergeometric test. The same clustering packages and parameters were used to visualize these genes as in Fig. 1a. An ANOVA was performed within each cluster to test for significant genotype*interaction effects with the aov() function in R. This approach ensured that DEGs were called as high-confidence HHO5-regulated N-dose genes in both mutants.

### Analysis of HHO5 direct binding and gene regulation

HHO5 TARGET RNA-seq data were acquired from ConnecTF.org (Brooks et al. 2021). Gene ontology analyses on these lists were performed as described for Fig. 1b. HHO5 DAP-seq and amp-DAP-seq datasets were generated by O’Malley et al. 2016. DAP-Seq reads were aligned to the TAIR10 genome, extended to the mean sequencing fragment size (200 BP), and normalized using an RPKM normalization. This resulted in bigwig files that were used as input into the R package EnrichedHeatmap to generate spatially resolved DAP-seq binding plots (Gu et al. 2018). Significance of the intersection between HHO5 binding and regulation was completed using the hypergeometric test in R. Cis-motif images were acquired from the Ecker lab plant cistrome database (O’Malley et al. 2016; Bartlett et al. 2017). Cis-motif enrichment analysis was completed in ConnecTF.org using the 500 BP promoters of each HHO target gene (Brooks et al. 2021).

### DoubleTARGET: genome-wide detection of gene regulation synergized by TF pairs

DoubleTARGET was carried out similarly to the standard TARGET approach (Bargmann et al. 2013). TFs were cloned into pBOB11 RFP or GFP vectors using Gateway Cloning. 3 to 4 million root protoplasts were transfected with 120 micrograms of plasmid DNA, which was prepped using the ZymoPure II Plasmid Maxi-prep kit (Zymo D4203). For single transfections (empty vector GFP (AKA EV), HHO5 alone as GFP, or WRKY21 alone as RFP), 120 µg of DNA was provided. For co-transfection samples, 60 µg of HHO5-GFP and 60 µg of WRKY21-RFP vectors were mixed and transfected in parallel with single transfection samples. After overnight incubation to allow for protein expression, cells were treated with 35 µM cycloheximide (CHX) for 20 min, followed by 2 h of 10 µM dexamethasone (DEX). Cells were suspended in 200 µL of W5 buffer supplemented with 0.01% BSA and successfully transfected cells were collected using a BD FACSDiscoveryS8. Ten thousand to 20,000 transfected cells were collected per sample and moved to dry ice. Four independent treatment replicates were completed per transfection condition. Cells were collected and frozen in RLT lysis buffer for later processing with the Qiagen RNeasy RNA micro prep kit (Qiagen 74004) with on-column DNAse digestion and quality checked using Agilent Tapestation HSRNA Kit (Aligent 5067-5579). mRNA was isolated using the NEB mRNA purification module (NEB E7490S). RNA was processed for sequencing using the NEB Ultra II library prep kit (NEB E7770S). Libraries were pooled and sequenced using an Illumina Novaseq 6000 S1 1 × 100 run.

The reads were basecalled using IlluminaBasecallsToFastq version 2.23.8, with APPLY_EAMSS_FILTER set to false (Broad Institute 2019). Following basecalling, the reads were demultiplexed using Pheniqs version 2.1.0 (Galanti et al. 2021). The entire process was executed using a custom nextflow pipeline, GENEFLOW (NYU CGSB, 2023). Reads were trimmed using CutAdapt (Martin 2011) to remove the standard illumina universal adapters. Trimmed reads were aligned to the Tair10 genome using STAR (Dobin et al. 2013). FeatureCounts from SubRead (Liao et al. 2014) was used to generate a count matrix that was used in DESEq2 (Love et al. 2014). DESEq2 normalized counts were used as input into the DEGPatterns clustering function from DEGReport (Pantano 2025). GO term enrichment was called within clusters using clusterProfiler in R (Yu et al. 2012).

### Network walking: linking HHO5 direct N-dose response targets to indirect N-dose response targets in a GRN

The HHO5 gene regulatory network (GRN) was generated using the network functions in ConnecTF. The 828 HHO5-regulated N-dose DEGs were used as input target genes, and edges were restricted to TARGET data (direct gene regulation) only for TFs that were both N-dose regulated and targets of HHO5 in both cells and *in planta*. TF2s acting downstream of HHO5 were then mapped to other N-dose responsive TF2s that were regulated by *HHO5 in planta*, but not in cells (indirectly regulated). The Network Walk was visualized using Cytoscape (Shannon et al. 2003). Genes identified as highly expressed in phloem were identified by scaling gene expression across cell types and subsetting for genes with a Z-score >1.

### Meta-analysis of inorganic and organic N-related genomic signals in planta

Data were gathered from Krouk et al. 2010b; Ristova et al. 2016; Goto et al. 2020; Swift et al. 2020, and organized into Table S9. Table S9 describes whether the dataset was RNA-seq, or microarray. Microarray probe IDs were converted using probe IDs from Lamesch et al. 2012. Three representative datasets were selected for intersection with the HHO5 GRN (Wang et al. 2004; Ristova et al. 2016; Goto et al. 2020). Hypergeometric tests were performed to test for intersection significance. The Sankey plots in Fig. 8 and Figure S19 were generated using the ggalluvium package in R (Brunson 2020).

## Results

Our study set out to investigate whether and how *HHO5*, a distinct member of the HHO TF family, mediates responses to N-dose in Arabidopsis. To this end, we hydroponically grew seedlings on low N media for 13 d (1 mM KNO_3_). After a day of N-starvation, we treated WT (Col-0) and 2 independent *hho5* T-DNA mutants *(hho5-1, hho5-2)* with each of 5 N-doses, ranging from 0.006 to 60 mM total N for 2 h (supplied as KNO_3_ + NH_4_NO_3_, see Materials and Methods). The highest N-dose reflected the amount of total N present in standard MS media (Murashige and Skoog 1962). We included a 0 and 20 mM KCl treatment to control for the effects of potassium in KNO_3_ (Figure S1). Two-hour exposure to the N-dose treatments ensured we could capture genes involved in early N-dose sensing. This experimental design was modeled on our previous studies, which revealed that *HHO5* is one of the earliest transcriptional responders to a temporal N-stimulus (Varala et al. 2018; Swift et al. 2020). Whole-root transcriptomes were analyzed with RNA-seq.

### N-dose treatments initiate inorganic and organic N signals in Arabidopsis roots

We first identified transcriptome-wide responses to N-dose in wild-type Col-0. Four thousand four hundred and sixty-four genes were N-dose responsive relative to the KCl control (Figure S2, Fig. 1). Of these, **1,702** genes were upregulated as N-dose increased, while **2,272** genes were downregulated (Fig. 1a and Table S1). Genes induced by N-dose were significantly enriched in gene ontology (GO) terms related to nitrate assimilation, glutamate metabolic process, hypoxia, and cytokinin responses. Previous studies also uncovered positive associations between N and cytokinin signals (Poitout et al. 2018). By contrast, genes repressed by N-dose were significantly enriched for defense-related terms, calcium transport, and water responses (Fig. 1b and Table S2). These N-dose repressed processes are also relevant to N-signaling (Riveras et al. 2015; Swift et al. 2019; Katz et al. 2022). Importantly, our N-dose treatments induced genes enriched for inorganic “nitrate assimilation,” as well as downstream “glutamate metabolic” organic N responses, meaning our N-dose treatments stimulated both inorganic and organic N-dose signals (Fig. 1).

As nitrate assimilation and glutamate metabolism GO Terms were enriched in the N-dose differentially expressed genes (DEGs), we next queried the N-dose response of individual genes in these pathways. Over half of the 59 KEGG N-metabolism genes (52.5%, Kanehisa et al. 2025) displayed significant gene expression responses to increasing N-dose (31/59, *P*-value = 2.90×10^−7^, Fig. 1c). For example, genes related to nitrate uptake (*NRT1*.*1*, *NRT2*.*1*), nitrite reduction (*NIA1*), and the assimilation of inorganic into organic N (*GS2, GLT1*, *GDH1*, *ASN1*, and *ASN2*) were upregulated in response to increasing N-dose. This is consistent with previous studies demonstrating N regulation of key N metabolism genes, including *NRT1.1* (Ho et al. 2009; Krouk et al. 2010a), *NRT2.1* (Nazoa et al. 2003; Wang et al. 2023), and *GLN1,3* (Moison et al. 2018). Organic N in the form of Glutamate (Glu), Glutamine (Gln), Aspartate (Asp), and Asparagine (Asn) is used to transport and store N (Lam et al. 1995). By contrast, genes previously shown to be responsive to N-starvation (*NRT2.4* and *GDH3)*, and ammonium transporters (*AMT1.1* and *AMT1.5*, Kiba et al. 2018), were downregulated in response to N-dose in our study. This analysis also distinguished N-metabolism genes induced specifically by N-starvation (0 mM N) from genes induced by extremely low N supply (0.006 mM N, Fig. 1c). Together, these results show that canonical inorganic and organic N-metabolism genes are sensitive to Total N treatments as a function of N-dose in Arabidopsis roots.

### *HHO5* responds to N-dose signals and is spatially diverged from the *NIGT1* TFs

**Figure 2:**
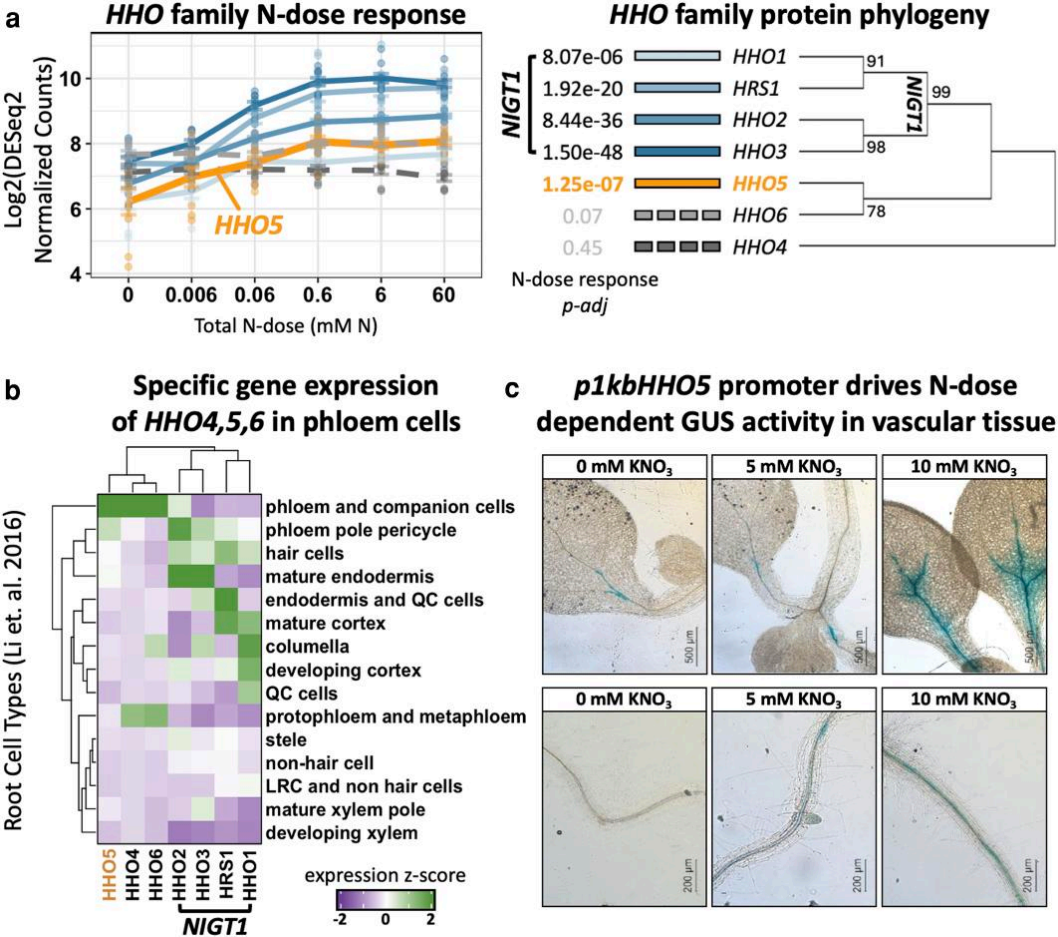
*HHO5* is a diverged, phloem-specific, and N-dose-responsive HHO TF. a) N-dose regulation of the 7 HHO family TF members (Log2 DESeq2-normalized gene expression). Error bars represent the standard error of the mean. Lines are colored by annotation in the HHO family protein phylogeny, annotated by the percentage of replicate trees in 1,000 bootstrap tests wherein branches cluster together. The HHO members are annotated by N-dose mRNA response, and significance is based on adjusted *P*-value from DESeq2. b) HHO TF expression quantified across the bulk RNA-seq root cell type atlas (Z-score scaled gene expression) from Li et al. 2016. c). Two-hour N-dose treatments enhance activity of p1kbHHO5::GUS expression in the vasculature of shoots and roots from 10-d-old plants across 3 N-dose conditions.

HHO5 was previously identified as an early N-response TF (Varala et al. 2018; Brooks et al. 2019). However, a potential regulatory role and mechanism for HHO5 in N-dose signaling has not yet been functionally characterized, compared with the NIGT1 TFs (Kiba et al. 2018; Safi et al. 2021). To fill this knowledge gap, we analyzed the N-dose response of the entire Arabidopsis HHO family in our RNA-seq experiment *in planta*. We found that 5 of the 7 HHO TFs significantly respond to N-dose treatments, including the 4 NIGT1 TFs and *HHO5* (Fig. 2a).

To investigate the spatial distribution of *HHO5* expression, we queried the gene expression of the HHO TF family across an Arabidopsis bulk RNA-seq root atlas (Li et al. 2016). Interestingly, the clustering of HHO TF gene expression across root cell types mimicked the HHO family protein phylogeny (Fig. 2b). The *NIGT1* TFs clustered together and were broadly expressed across root cell types. By contrast, the diverged HHO TFs (*HHO4,5,6*) displayed a more specific pattern, expressed predominantly in the phloem and companion cells. Notably, *HHO5* clustered away from *HHO4* and *HHO6*, as *HHO5* is not expressed in the protophloem or metaphloem. Instead, *HHO5* is expressed in the phloem pole pericycle, an important location for protein and solute unloading in roots (Ross-Elliott et al. 2017) (Fig. 2b).

To validate the spatial expression pattern of *HHO5*, we conducted GUS reporter activity experiments using the 1 kb promoter upstream of *HHO5* (*p1kbHHO5*). We found that *p1kbHHO5* driven GUS activity increased in response to N-dose in shoots and roots, and was localized to the vascular tissues (Fig. 2c). In further support of the phloem localization of *HHO5*, we note that a recent single-cell RNA-seq study identified HHO5 as a core foundational TF significantly linked to phloem cell (*P*-adj = 5.96×10^−233^), and companion cell identity (*P*-adj < 1×10^−250^) (Xue et al. 2025). Another study found phloem and companion cell-specific enrichment of the Arabidopsis HHO5 DAP-seq cis motif in pearl millet (Yan et al. 2026). Therefore, the phloem specificity of HHO5 may be found in other species as well. In summary, *HHO5* is a distinct HHO TF, as its gene expression responds significantly to N-dose treatments, and is preferentially expressed in phloem. This positions a key role for HHO5 in N-dose signaling, as the phloem is an important conduit for inorganic and organic N uptake and transport.

### HHO5 regulates the amplitude of transcriptome-wide N-dose responses *in planta*

**Figure 3:**
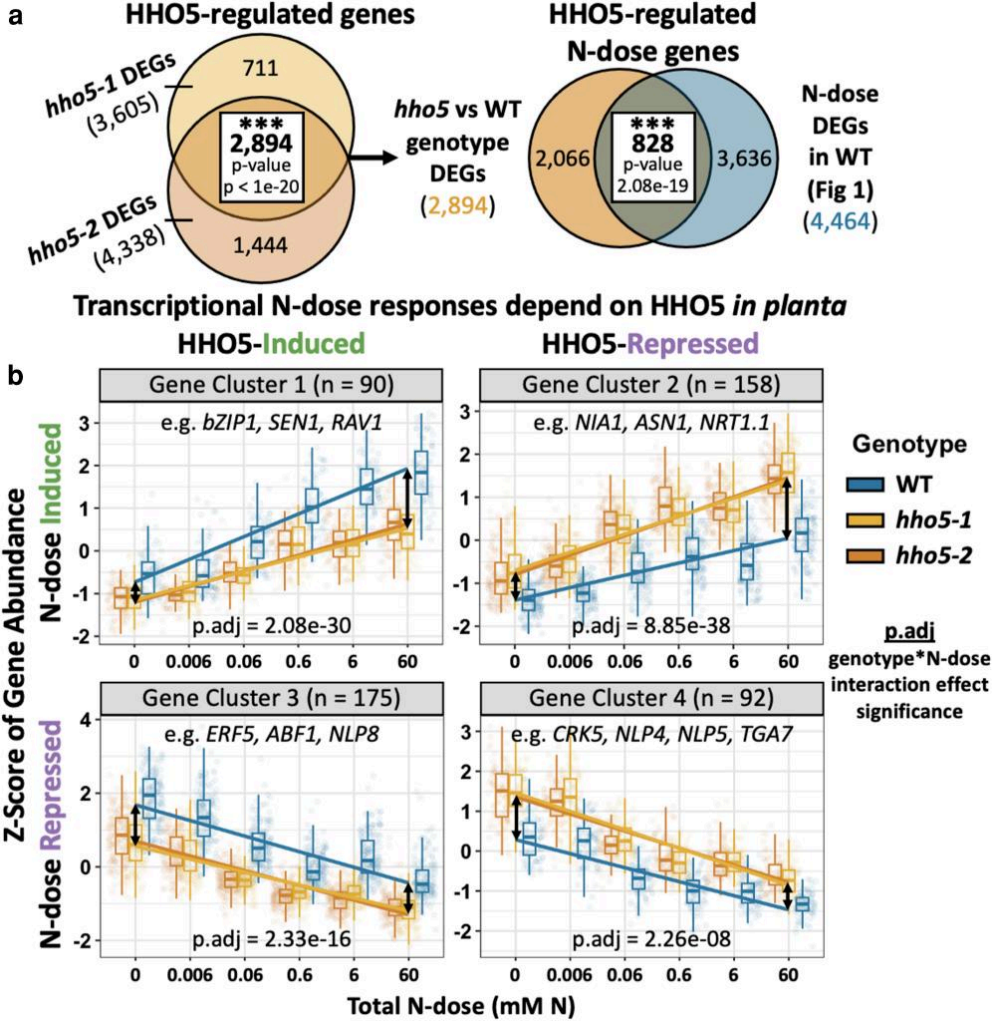
HHO5 regulates the amplitude of N-dose responses *in planta.* a) Intersection of DEGs (hho5 vs WT) from 2 hho5 T-DNA mutants (DESeq2 *P*.adj < 0.05) for the genotype effect. Intersection significance is the result of a hypergeometric test. Two thousand eight hundred and ninety-four genes are regulated in both hho5 mutant alleles, 828 of which intersect with the 4,464 N-dose DEGs from Fig. 1. b) Expression profiles of 4 largest clusters of HHO5 N-dose DEGs. Synergistic N-dose * genotype effects are quantified by ANOVA on z-score scaled data, where a significant interaction effect was detected and shown at the bottom of each plot.

Our next goal was to evaluate the role of HHO5 in mediating plant N-dose responses. We found that mutating *HHO5* resulted in misregulation of hundreds of N-dose genes *in planta* (Fig. 3). First, we used data from the 2 *hho5* T-DNA mutants in our N-dose RNA-seq experiment (Figure S1) to identify HHO5 target genes *in planta,* relative to the WT control. To ensure we found high-confidence *hho5* DEGs, we only considered a gene to be regulated by HHO5 if it was significantly perturbed in both *hho5* mutant alleles. We found **2,894** DEGs were shared between the 2 *hho5* mutant backgrounds, representing a highly significant DEG overlap (Fig. 3a, Figure S3a and b and Table S3). Moreover, we found that the log2 fold change (mutant/WT) for the shared DEGs were highly correlated between the 2 *hho5* mutant alleles (R^2^ = 0.99, *P*-value < 2.2×10^−16^, Figure S3c). Thus, we robustly identified thousands of genes significantly regulated by HHO5 *in planta*.

Importantly, we found that a significant portion of the HHO5 target genes *in planta* also respond to N-dose treatments (828 genes, *P* = 2.08×10^−19^, Fig. 3a and Table S4). HHO5 therefore regulates a substantial fraction (∼19%) of the N-dose responsive transcriptome detected in wild-type plants. To visualize the N-dose DEGs regulated by HHO5, we clustered and plotted the expression of the DEGs across genotypes (WT, *hho5-1, hho5-2*) and N-doses (0 to 60 mM) (Fig. 3b and Figure S4). The majority of the genes regulated by HHO5 fell into 4 clusters, which were either induced or repressed by HHO5 and N-dose (Fig. 3b).

For example, genes in Cluster 1 increase expression with increasing N-dose in WT (blue line). However, the expression of Cluster 1 genes was lower in *hho5* mutants (orange lines) relative to WT (blue line). Therefore, Cluster 1 N-dose response genes are induced by HHO5 *in planta.* These DEGs include other TFs involved in N-responses, including *bZIP1*, which was identified as a hub of an organic N-network in Arabidopsis (Gutiérrez et al. 2008). In contrast, the expression of genes in Cluster 2 was repressed by HHO5. Cluster 2 included genes involved in N-uptake, sensing, and transport including *NRT1.1, NIA1*, and *ASN1* (Fig. 3b). HHO5 repression of genes involved in N-uptake/reduction is consistent with previous literature stating that HHO TFs act as repressors of nitrate uptake (Safi et al. 2021). However, unlike other studies of HHO TFs, we found that HHO5 also induces the expression of many N-dose response genes (Clusters 1 and 3, Fig. 3b).

To determine if transcriptional N-dose response amplitudes were perturbed in *hho5* mutants, we used an ANOVA to test for a genotype*N-dose interaction effect within these gene clusters. The ANOVA revealed that the amplitude of N-dose response in all 4 clusters, on average, significantly depends on the expression level of *HHO5*. Therefore, depending on the target, we found that HHO5 can either induce or repress the amplitude of N-dose response for hundreds of genes *in planta* (Fig. 3).

### HHO5 promotes N-dose dependent phenotypic growth *in planta*

**Figure 4:**
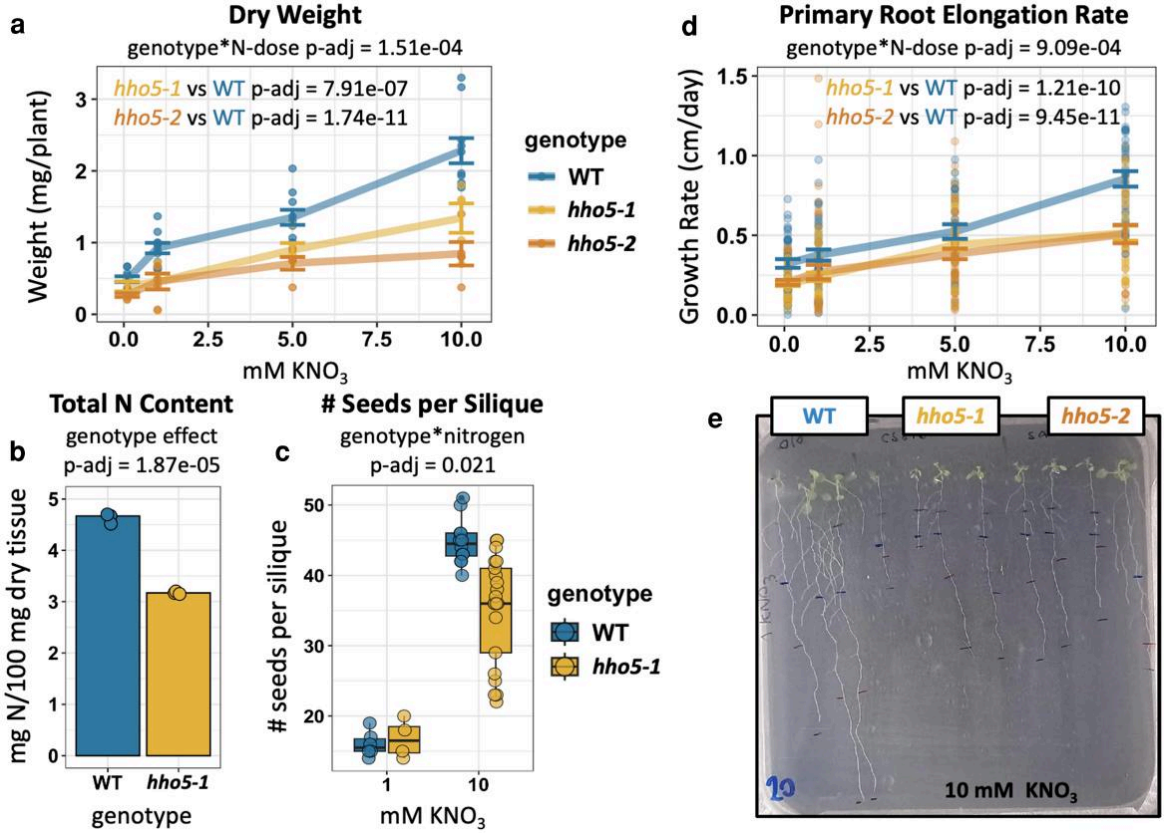
*hho5* mutants display significantly dampened N-dose responses *in planta.* a) Plant dry weight measured across N-doses in WT and hho5 mutants, where an ANOVA was performed to test for a significant genotype*N-dose interaction effect. Reproductive traits are compromised in hho5 mutants, compared to WT, where b) total N content in seeds of plants grown with 10 mM KNO_3_, and c) the number of seeds per silique, are significantly decreased in the hho5-1 mutant compared to WT plants. d) Primary root growth rate (cm growth/day) varies significantly across N-dose, and this is significantly perturbed in both hho5 T-DNA mutant alleles. e) Image of root lengths at 10 mM KNO_3_. This phenotype was also confirmed on NH_4_NO_3_, and Total N supplemented media, see Figure S5. Error bars represent the standard error of the mean.

We next measured the impact HHO5 has N-dose dependent Arabidopsis growth *in planta*. To do this, we compared N-dose dependent growth of *hho5-1* and *hho5-2* mutants to wild-type seedlings (Col-0), where all treatments were performed on nutrient media identical in composition except for N concentration (see Materials and Methods for details). These plants were grown on 4 different N-doses that ranged from a growth-limiting dose of 0.1 mM to a moderate dose of 10 mM KNO_3_. For these long-term growth experiments, KNO_3_ media is expected to induce both inorganic and organic N responses. As expected, we found wild-type plants were plastic in response to varying N-doses. Specifically, both plant dry weight and primary root length significantly increased with N-dose (*P* = 1.06×10^−12^ for dry weight, *P* < 2.0×10^−16^ for primary root length, Fig. 4).

However, the plant's ability to respond to N-dose was impaired in both *hho5* mutant alleles. We uncovered a significant reduction in shoot dry weight that intensified with increasing N-dose, which was indicated by a significant genotype*N-dose interaction effect (Fig. 4a). Therefore, growth of *hho5* mutants is impaired in an N-dose-dependent manner. The *hho5-1* mutant also displays a significant reduction in seed N content, and a reduction in the number of seeds per silique (Fig. 4b and c). This suggests that HHO5 may be important for promoting the transport of organic N in the phloem (Fig. 2c) to developing tissues such as seeds. We also confirmed these phenotypic differences were also detected in response to growth on 4 doses of ammonium nitrate, and combinations of nitrate and ammonium nitrate (Total N) (Figure S5). Overall, these studies demonstrate that HHO5's role in mediating N-dose responses at the transcriptional level (Fig. 3) has a strong impact on plant phenotypes.

We previously found that N-dose responses are dynamic, and that perturbing the TF *TGA1* affects N-dose responses according to Michaelis–Menten (MM) kinetics (Swift et al. 2020). Our prior study showed that *HHO5* is a target of TGA1 (Swift et al. 2020), and that *HHO5* mRNA levels respond to N-dose according to MM kinetics. Thus, we sought to test whether N-dose dependent growth *rates* in Arabidopsis are also controlled by HHO5. To test this, we grew WT and *hho5* mutant seedlings across 4 N-doses of KNO_3_, and measured the primary root length at 3-d intervals across 4 time-points (Figure S6). In WT, the rate of primary root growth significantly increases with increasing N-dose (*P* < 2×10^−16^). By contrast, N-dose dependent primary root growth rates were significantly perturbed in both *hho5* mutant alleles (Fig. 4d). Therefore, HHO5 is required for maintaining primary root growth rates in response to N-dose (Fig. 4e).

### The direct targets of HHO5 in plant cells are enriched for genes responding to inorganic and organic N signals

The finding that *hho5* mutants display a significant reduction in N-dose-dependent phenotypes was unexpected, given the canonical repressive role of the redundant NIGT1 TFs (Kiba et al. 2018). Quadruple mutants of the redundant *NIGT1* TFs were previously shown to display increased plant growth in high N conditions (Safi et al. 2021). HHO5 is thus diverged phylogenetically, spatially, and in our finding that HHO5 can induce N-dose-dependent genes and phenotypes *in planta* (Figs. 3 and 4). To better understand the role of HHO5 in regulating N-dose-dependent gene expression and phenotypes, we sought to characterize the HHO5 regulatory mode of action.

To this end, we set out to identify the directly regulated target genes of HHO5. We first mined published HHO5 direct target genes detected using the cell-based TARGET TF perturbation assay (Varala et al. 2018; Brooks et al. 2023). In TARGET, a GR:TF fusion is transiently over-expressed in plant protoplasts (Bargmann et al. 2013). Dexamethasone (DEX) treatment facilitates GR:TF nuclear import, allowing for precise control of experimental timing. Crucially, prior to DEX-treatment, the addition of cycloheximide (CHX) prevents translation of regulated transcripts downstream of the GR:TF fusion (Yamaguchi et al. 2015). These GR:TF regulated transcripts can include downstream TF2s that would propagate signals if not for translation inhibition by CHX. Thus, TARGET RNA-seq identifies genes directly regulated in response to GR:TF nuclear import relative to an empty vector (EV, no TF control) (Brooks et al. 2023).

**Figure 5:**
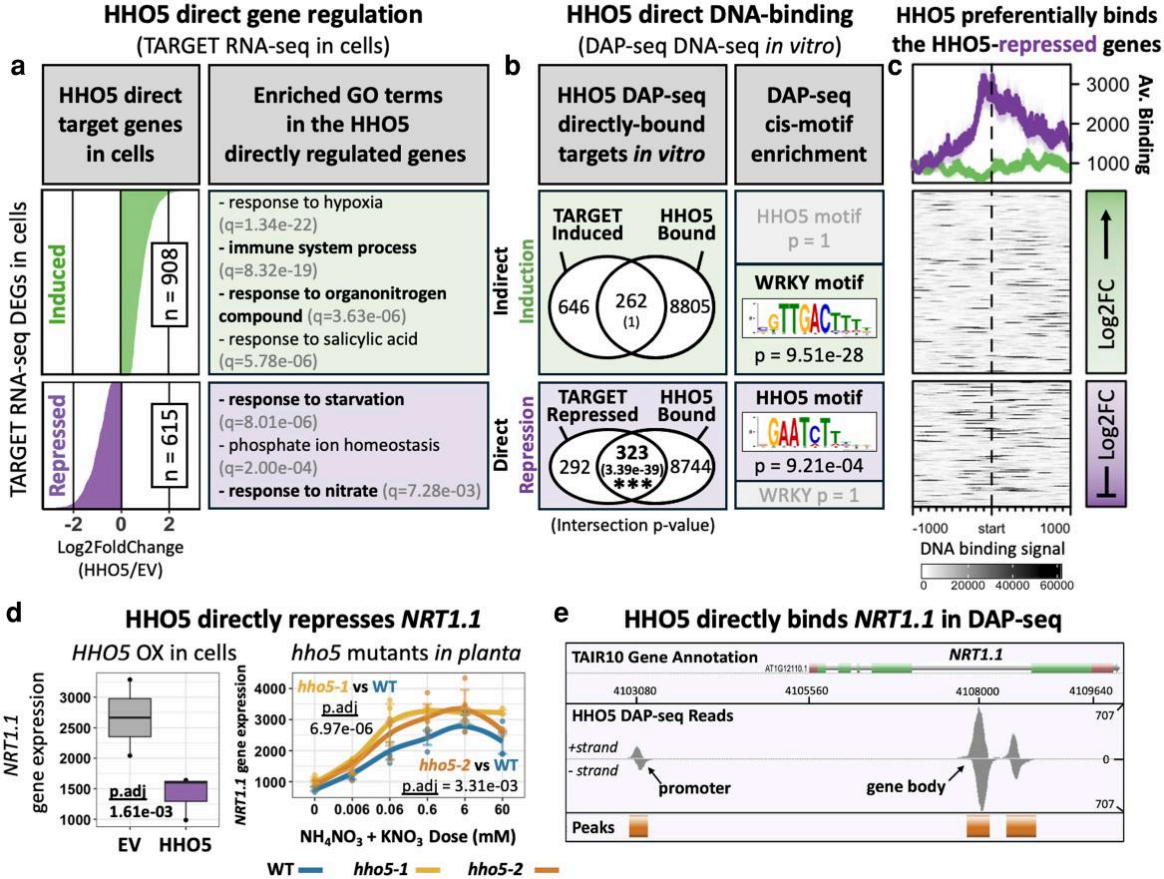
HHO5 directly represses nitrate response genes and indirectly induces genes responsive to organonitrogen compounds. a) HHO5 directly regulated target genes identified in shoot cells from the TARGET assay and associated GO Terms, with q-values for enrichment significance. b) HHO5 DAP-seq direct TF-DNA binding (O’Malley et al. 2016) intersected with directly regulated DEGs from TARGET assay (*P*-values from a hypergeometric test). c) HHO5 DAP-seq signal plotted over the HHO5 TARGET directly regulated genes. The average signal for the induced and repressed genes plotted across the gene sets. The HHO5 target genes are ordered by Log2 fold change (GR:HHO5/GR:EV) and present +/− 1 kb of sequences around the gene transcription start sites. d) NRT1.1 expression in TARGET (Varala et al. 2018) and in planta (this study, Fig. 1). Error bars represent the standard error. e) HHO5 DAP-seq DNA Binding from the Ecker lab genome browser (Bartlett et al. 2017).

Analysis of the TARGET data reveals that HHO5 directly induces **908** genes and directly represses **615** genes in Arabidopsis shoot protoplasts (Varala et al. 2018, Fig. 5a, Table S5). This finding reinforces the notion that HHO5 can either induce or repress expression, depending on the target gene. GO term analysis revealed that genes directly induced by HHO5 were significantly enriched for organic N response, defense, and hypoxia; terms related to the genes induced by N-dose *in planta* (Table S6). By contrast, genes directly repressed by HHO5 are enriched in responses to nutrient starvation, nitrate, and phosphorus (Table S6). Therefore, the direct target genes of HHO5 in cells (target genes directly downstream of HHO5) encompass both inorganic and organic N responses (Fig. 5a).

HHO5 directly induces genes enriched in organonitrogen compound responses (Fig. 5a). The organonitrogen response genes directly induced by HHO5 include a significant number of the glutamate receptor-like (GLR) genes (6/20, *P* = 1.81×10^−4^, Chiu et al. 2002, Figure S7). In addition, **14/18** of the organonitrogen response genes that are induced by HHO5 in TARGET were also induced by HHO5 *in planta* (eg N-dose response significantly lower in the *hho5* mutants compared to WT, Figure S7a and b). Interestingly, these 14 organonitrogen response genes are uniquely induced targets of HHO5. More specifically, organonitrogen-related direct targets of HHO5 are generally not regulated, or in a few cases are repressed, by the NIGT1 TFs with available data. This was determined by mining TARGET data for the NIGT1 TFs in ConnecTF.org (*HHO2, HHO3*, Brooks et al. 2021, Figure S7c). Therefore, *HHO5* is further separated from the NIGT1 TFs, in that it directly induces genes related to organonitrogen signaling (Fig. 5).

### HHO5 is a direct repressor and indirect inducer of gene expression

We next sought to determine which HHO5 target genes are directly bound by HHO5. To this end, we characterized HHO5 in vitro DNA-binding using published DAP-seq data (O’Malley et al. 2016). In DAP-seq, the TF of interest is fused to a HALO tag and expressed in vitro. After incubation with a genomic DNA library, affinity-purification of the HALO:TF fusion captures genome-wide DNA-binding. As no other TFs are expressed in vitro, DAP-seq signal represents direct binding of the TF to DNA (Bartlett et al. 2017). Interestingly, we found significant overlap between the HHO5 DAP-seq bound genes and the HHO5-repressed genes. However, no such enrichment of HHO5 DNA-binding was observed among genes induced by HHO5 in plant cells (Fig. 5b, Table S7). This suggests that direct DNA binding by HHO5 is associated with gene repression, as has been previously found for HHO5 and the NIGT1 TFs (Moreau et al. 2016; Kiba et al. 2018; Ueda et al. 2020).

We further explored the mode-of-action for HHO5 gene interactions using cis-element-enrichment analysis (Fig. 5b). We found the canonical HHO5 DNA-binding motif from DAP-seq is significantly enriched in the HHO5-repressed genes (Fig. 5b). Unexpectedly, we found that the genes directly induced by HHO5 in TARGET are not enriched in the HHO5 cis element but instead are significantly enriched for the WRKY TF family-binding motif (Fig. 5b). To better understand where HHO5 is binding its target genes, we plotted HHO5 DAP-seq DNA-binding signal over all HHO5 regulated genes from the TARGET assay. The HHO5 DAP-seq signal was stronger among the HHO5-repressed genes and localized to promoters and transcription start sites (labeled as start, Fig. 5c). In total, HHO5 directly binds the promoters of the HHO5-repressed genes, and HHO5 target gene induction may instead be mediated by indirect DNA binding (Fig. 5b and c).

As an example, target gene directly bound and repressed by HHO5, we show *NRT1.1,* the *“nitrate transceptor”,* a key gene involved in nitrate uptake and sensing (AT1G12110, Bouguyon et al. 2015). Specifically, *NRT1.1* is repressed by HHO5 both in cells and *in planta* (Fig. 5d). We also found that HHO5 directly binds to the promoter and gene body of *NRT1.1* in DAP-seq (Fig. 5e). We repeated these analyses for other HHO TF family members with available data (O’Malley et al. 2016) and found the same relationship between direct DNA binding and HHO5-target gene repression (Figure S8). Therefore, direct repression of nitrate responses is conserved between HHO5 and the NIGT1 TFs (Kiba et al. 2018; Ueda et al. 2020).

**Figure 6:**
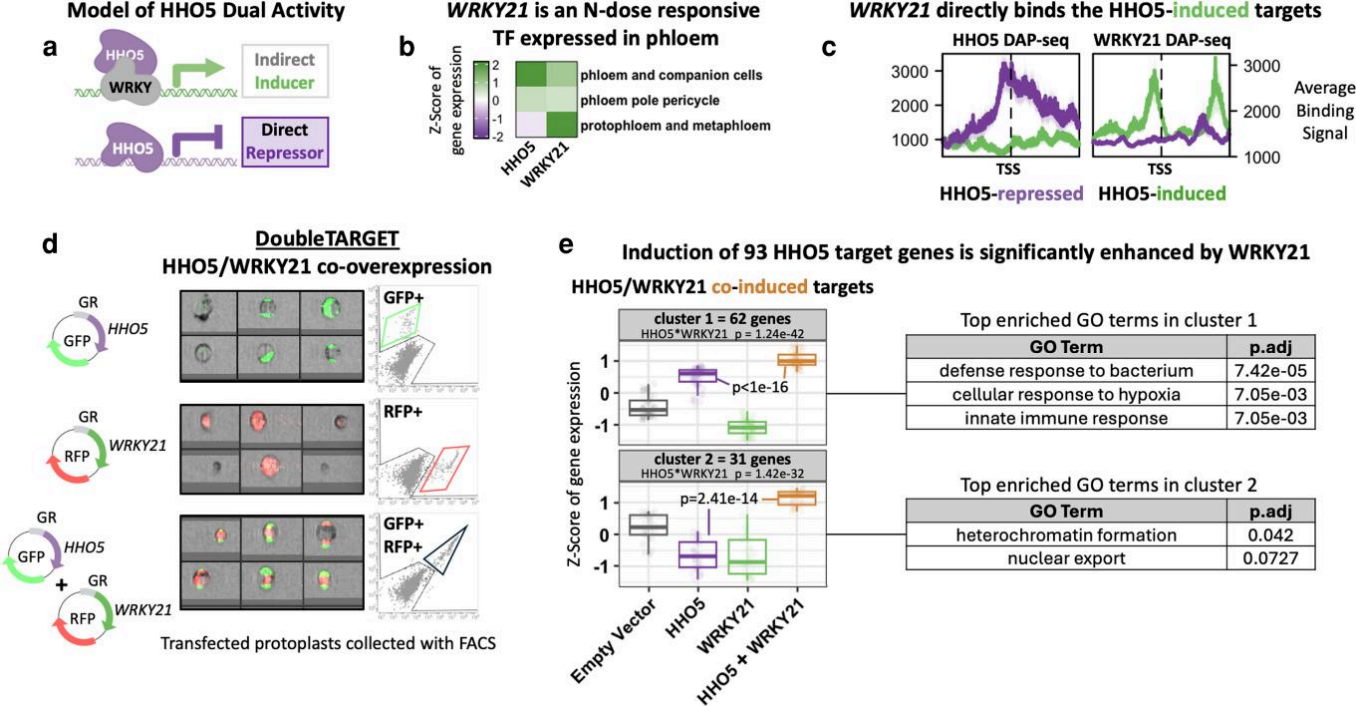
WRKY21 is a putative partner TF that enhances HHO5 induction of defense genes in plant cells. a) HHO5 directly binds and represses targets, while we propose that HHO5 indirectly binds and induces target genes via WRKY partner TFs (Fig. 5). b) The putative partner WRKY21 is expressed in phloem cells along with HHO5. c) DAP-seq (O’Malley et al. 2016) DNA binding profiles for HHO5 and WRKY21 near the HHO5 target gene transcription start sites (TSS). d) Schematic of DoubleTARGET, in which HHO5 and/or WRKY21 were transiently expressed in Arabidopsis root protoplasts. GFP/RFP DoublePositive cells over-expressing both HHO5 and WRKY21 were isolated using FACS. Images of successfully transfected (or co-transfected) protoplasts taken by a BD FACSDiscoverS8 are shown next to representative distributions of cells from different gating conditions. e) Ninety three of the validated HHO5-induced target genes from Fig. 5 are expressed significantly higher in cells that co-overexpress WRKY21 with HHO5. Significant GO Term enrichment from both clusters are shown.

Next, we sought to understand the mechanism by which HHO5 induces target genes. One hypothesis is that direct induction of target genes by HHO5 may be due to *indirect* HHO5 DNA binding via partner TFs (Fig. 6a). This hypothesis is supported by the enrichment of WRKY family motifs in the HHO5-induced genes, and corresponding enrichment of WRKY-associated GO terms such as “immune system process” (Pandey et al. 2009, Fig. 5b and c). Therefore, WRKY TFs may help facilitate the binding of HHO5 to its induced target genes (Fig. 6a).

### WRKY21 is a putative partner TF that enhances HHO5 target gene induction

We next sought to validate the hypothesis that WRKY partner TFs mediate HHO5 target gene induction. As there are 72 WRKY TFs in Arabidopsis (Cheng et al. 2017), we prioritized possible WRKY partner TFs based on their N-dose response and gene expression levels in phloem, as shown for HHO5 (Figure S9). We reasoned that WRKY TFs that are spatially and conditionally co-expressed with *HHO5* are more likely to act as HHO5 partner TFs. We found that 28 of the 72 WRKY TFs display significant gene expression responses to N-dose in our WT dataset (Fig. 1). Of these 28 N-dose responsive WRKY TFs, 8 displayed relatively high expression in phloem (Figure S9).

Of these 8 phloem-expressed N-dose responsive WRKY TFs, *WRKY21* was of special interest as a possible HHO5 partner TF. We previously found that *HHO5* and *WRKY21* are both transcriptionally induced specifically after 10 min of N treatments in a validated temporal network of dynamic N responses (Fig. 3 in Varala et al. 2018). Therefore, *WRKY21* is an N-dose-responsive TF that is spatially and temporally co-expressed with *HHO5* (*WRKY21* N-dose response *P*-adj = 1.40×10^−3^) (Fig. 6b). In further support of WRKY21 acting as a partner TF of HHO5, we plotted WRKY21 DAP-seq binding and found that unlike HHO5, WRKY21 displays direct binding specifically to the HHO5 induced genes (O’Malley et al. 2015, Fig. 6c). This suggests specificity in WRKY21's putative role as a partner TF that facilitates HHO5 target gene induction (Fig. 6c).

To experimentally validate the hypothesis that WRKY21 aids HHO5 in target gene induction, we adapted the plant cell-based DoubleTARGET assay (Fig. 6d). DoubleTARGET measures the combinatorial effects of TF pairs, genome-wide. In DoubleTARGET, separate overexpression vectors each carrying one of the 2 TFs of interest are co-transfected into the same protoplasts with either RFP or GFP fluorophore labels. Fluorescence activated cell sorting (FACS) allows for the specific collection of cells that display both RFP and GFP fluorescence and thus co-overexpress both TFs of interest (Figure S10, Fig. 6d). We then use RNA-seq to analyze the genome-wide effects of TF co-overexpression relative to single TF and empty vector (EV) transfection controls (Fig. 6d).

We previously found that HHO5 induces 908 target genes that are enriched for organonitrogen and defense responses using the standard TARGET assay (Varala et al. 2018, Fig. 5). These targets were identified by overexpressing *HHO5* in cells wherein putative partner TFs are present, but only at endogenous expression levels. With DoubleTARGET, we asked if co-overexpression of the putative partner TF *WRKY21* with *HHO5* enhanced the induction of any of these 908 HHO5 induced targets (Figure S11). We found that 93 (∼10%) of the targets were expressed significantly higher when WRKY21 was co-overexpressed with HHO5, compared to cells only over-expressing HHO5. Interestingly, WRKY21 alone tended to repress these 93 genes, so the specific combination of HHO5 and WRKY21 is what led to increased expression of these targets. The HHO5/WRKY21 co-induced genes were enriched for defense related signaling (Fig. 6e). Therefore, WRKY21 stands out as a putative partner TF that aids HHO5 in the induction of defense signaling in plant cells. Together, these results support a model in which HHO5 functions as an indirect gene activator by partnering with WRKY TFs.

We generated preliminary evidence that WRKY21 is one, of likely many, putative partner TFs of HHO5. Partner TFs therefore could mechanistically explain how HHO5 induces target genes. However, we also hypothesized that induction of some HHO5 targets could be mediated by secondary TF2s directly downstream of HHO5 (HHO5-TF2s). We chose to explore this TF2 hypothesis below using a Network Walking approach.

### Network walking links HHO5 to its direct and indirect targets which comprise 12% of the N-dose-responsive transcriptome *in planta*

Our RNA-seq experiments showed that HHO5 regulates 828 N-dose response genes *in planta* (Fig. 3a). We next sought to determine which of these 828 N-dose response genes were direct or indirect targets of HHO5. To this end, we deployed a “Network Walking” approach described in (Brooks et al. 2021; Huang et al. 2023). This approach enabled us to identify a network path between HHO5 direct targets identified in cells and HHO5 targets identified *in planta* via HHO5-TF2s, as described below.

**Figure 7:**
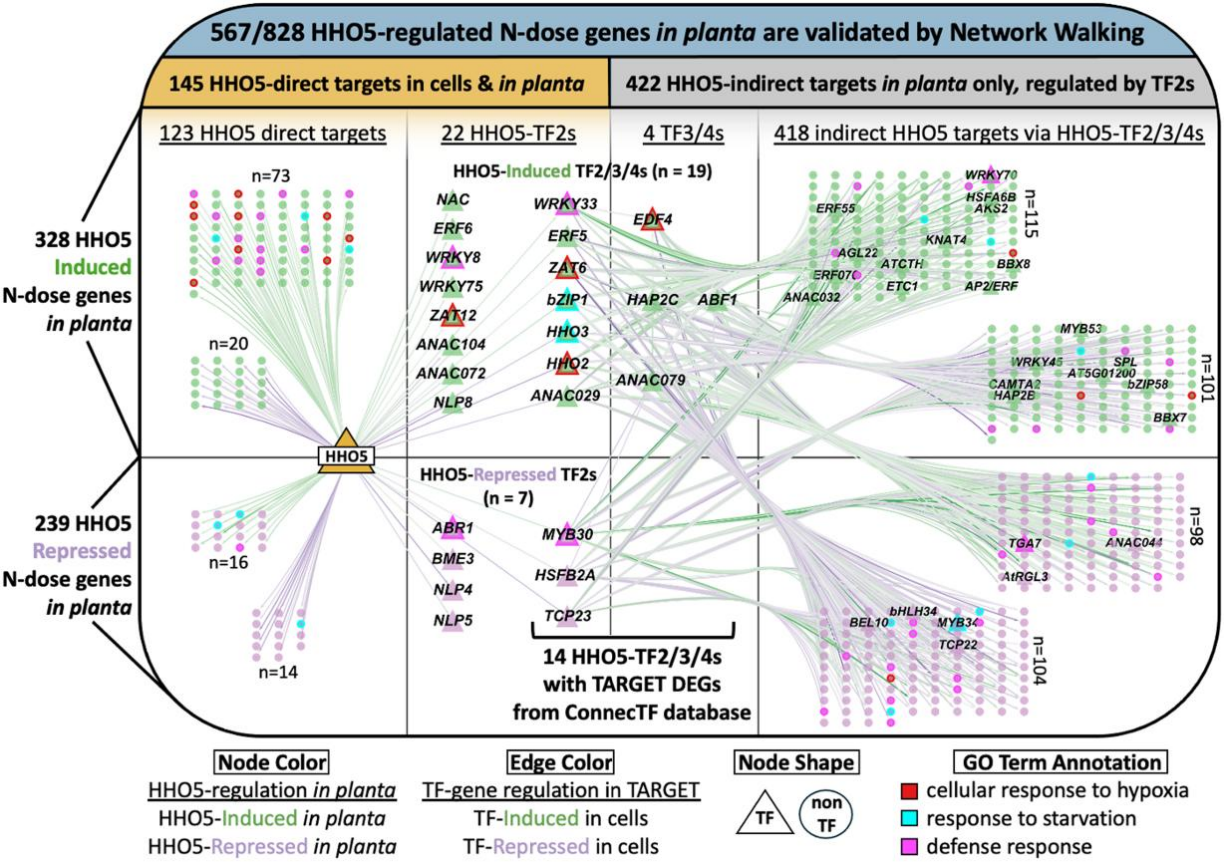
Network walking: a network path links HHO5 direct and indirect targets to 12% of the total N dose response genes *in planta.* The functionally validated Gene Regulatory Network (GRN) is composed of 567 nodes which represent N-dose response genes regulated by HHO5 in planta (Fig. 3). Green nodes denote genes induced by HHO5 in planta, and purple nodes show genes repressed by HHO5 in planta. All edges shown are TF-gene direct regulatory relationships, validated in the cell based TARGET assay, where green edges denote gene induction and purple edges denote gene repression (Fig. 5). TF nodes are shown as triangles, and are labeled by gene name.

To chart the HHO5 network path, we first used the direct targets of HHO5, detected using the TARGET cell-based assay. We first identified which of the HHO5-dependent N-dose response genes *in planta* were direct HHO5 targets (Fig. 3a, Fig. 5). This analysis revealed that **145/828** N-dose response genes are regulated by HHO5 both in cells (TARGET assay) and *in planta* (T-DNA mutants). These 145 *bona fide* direct targets of HHO5 include **22** TF2s (eg HHO5-TF2s), and are functionally enriched for response to N compound (q = 5.92×10^−4^), response to hypoxia (q = 6.29×10^−7^), and response to starvation (q = 1.34×10^−2^) (Figure S12, Fig. 7). Thus, HHO5 directly regulates N-dose response genes related to N-response and nutrient starvation both in cells and *in planta*. As only 145/828 N-dose-response genes misregulated in *hho5* mutants could be explained by HHO5 direct gene regulation, we reasoned that the 22 directly regulated TF2 targets of HHO5 could potentially regulate the remaining HHO5 indirect N-dose response target genes.

To test if HHO5 initiates a signaling cascade of N-dose response genes via HHO5-TF2s, we leveraged TARGET data for Arabidopsis TFs housed in the ConnecTF database (ConnecTF.org, Brooks et al. 2021). We found that **10/22** HHO5-TF2s had validated genome-wide direct targets in the ConnecTF database. In addition, 4 TFs downstream of the HHO5-TF2s also had TARGET data in ConnecTF. These were designated as TF3s (3 TFs) and one TF4 was identified downstream of a TF3. Validated direct targets from these 14 TF2/3/4s acting downstream of HHO5 further explained the misregulation of 418/828 N-dose response genes in *hho5* T-DNA mutants *in planta* (Table S8). We visualized these validated direct and indirect regulatory interactions downstream of HHO5 and the HHO5-TF2/3/4s in a gene regulatory network path (GRN, Fig. 7, Figure S13).

In total, **567/828** (68.5%) of the HHO5 regulated N-dose response genes *in planta* were functionally validated using TARGET gene regulation data for HHO5 and its downstream TFs (HHO5-TF2s, 3s and 4s). This network validation of HHO5 direct and indirect targets encompasses ∼**12%** of the total N-dose response genes detected in wild-type plants (a significant intersection, *P* = 1.98×10^−18^). The genes induced by HHO5 in this validated HHO5-GRN were enriched for the glutamate metabolism related term “cellular response to acid chemical” (q = 2.6×10^−2^) and hypoxia (q = 7.5×10^−6^). By contrast, the genes repressed by HHO5 in the GRN are enriched for inorganic anion transport (q = 0.02) and nitrate import (q = 0.04). Therefore, these results suggest that HHO5 induces expression of genes responding to organic N signals and represses genes whose expression responds to inorganic N signals, both in cells and *in planta*.

Intriguingly, some of the validated targets in the N-dose response GRN path downstream of HHO5 are expressed highly in phloem. We found **64** HHO5 targets validated in this network path that are expressed highly in phloem cells (Z-score = >1, Figure S14a). These validated HHO5 target genes expressed in phloem are enriched for functions related to nitrate import, regulation of root development, and response to starvation (Figure S14b). We previously showed that HHO5 repressed nitrate responses. Our cell-type specific analysis of the HHO5 GRN suggests this repression may occur in the phloem, where nitrate is transported (Fig. 5). With regard to organic N dose responses, we found that HHO5 directly induces *bZIP1* expression in cells and *in planta* (Fig. 7*)*. *bZIP1* is an established master regulator of organic N signaling (Gutiérrez et al. 2008; Para et al. 2014), which we found is also expressed in phloem cells (Figure S14c). Thus, the validated network path for HHO5 determined using the Network Walking approach provides evidence to support a role for HHO5 in regulating inorganic and organic N-dose dependent response genes in phloem.

In total, our genomic analyses show that HHO5 can either directly repress or indirectly induce genome-wide N-dose responses. We provide experimental evidence supporting 2 different mechanisms of indirect target gene induction by HHO5. First, WRKY partner TFs, such as WRKY21, may co-induce target genes with HHO5 (Fig. 6). Secondly, we uncovered 22 HHO5-TF2s, which may propagate N-dose signals downstream of HHO5 using network walking (Fig. 7). In support of this, we found that *WRKY33*, a glutamate-induced direct TF2 target of HHO5, induces 8/18 organonitrogen genes discussed above, and strongly binds the HHO5-induced gene set, based on DAP-Seq in vitro binding (Figures S7 and S8). HHO5 is therefore a validated dual regulator of organic and inorganic N-dose responses, via direct repression, or indirect target gene induction via partner TFs and HHO5-TF2s.

### *HHO5* expression is induced by organic N signals, but repressed by inorganic N signals

The finding that HHO5 can either induce or repress genome-wide N-dose-responses adds new insights into the role that HHO5 may play in regulating responses to inorganic and organic N signaling. This led us to question whether *HHO5* gene expression itself responds to inorganic or organic N signals, and how this may influence the HHO5 regulatory mode-of-action. To address this, we conducted a meta-analysis that integrated multiple genomic datasets related to inorganic and organic N signaling (Wang et al. 2004; Gutiérrez et al. 2008; Hu et al. 2009; Krouk et al. 2010b; Bouguyon et al. 2015; Ristova et al. 2016; Muñoz-Nortes et al. 2017; Varala et al. 2018; Goto et al. 2020; Swift et al. 2020; Liu et al. 2022b; Monden et al. 2025) (Table S9).

**Figure 8:**
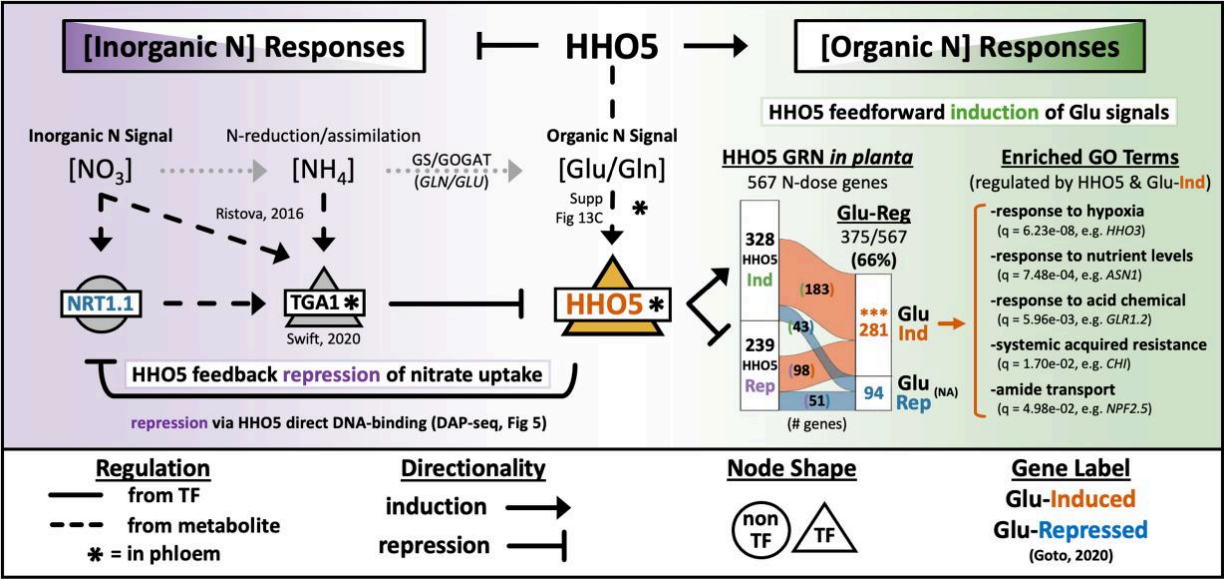
HHO5 orchestrates feedback regulation between organic and inorganic N-dose dependent signaling in Arabidopsis. Top: schematic of inorganic N input assimilated into organic-N amino acids for N-assimilation (Glu/Gln). Inorganic-N (nitrate) signaling: sensing and transduction of the nitrate signal by NRT1.1 and downstream master regulator TGA1 are both induced by nitrate (Ristova et al. 2016). TGA1 represses the Michaelis-Menten N-dose response of HHO5 (Swift et al. 2020). Organic N signaling: organic N induction of HHO5 expression feedback represses expression of NRT1.1, Glutamate induces HHO5 expression (Gutiérrez et al. 2008; Muñoz-Nortes et al. 2017; Goto et al. 2020) (Figure S15). HHO5 in turn initiates a validated network path of 567 N-dose response genes in planta (Fig. 7). A significant portion (*P* = 1.72×10^−25^) of this validated HHO5 GRN is induced in response to glutamate treatment (Goto et al. 2020). The HHO5 targets that are induced by glutamate treatment in planta (Goto et al. 2020) are enriched for GO terms related to nutrient signaling and defense.

In constructing this model, we first asked which N species and doses stimulate *HHO5* gene expression. Our N-dose RNA-seq experiment performed *in planta* utilized total inorganic N treatments (NH_4_NO_3_ + KNO_3_), which evokes responses to inorganic N (nitrate, ammonium), and their primary assimilation into organic N products (Glu/Gln; Asp/Asn) (Fig. 1). *HHO5* gene expression was induced under the N-dose treatments (0 to 60 mM Total N) used in our study (Figure S15a). *HHO5* showed similar induction responses in our prior studies of time-based Total N-treatments of Arabidopsis (Varala et al. 2018; Swift et al. 2020). By contrast, *HHO5* gene expression was *not* significantly regulated by separate nitrate or ammonium treatments, when given in low doses (1 mM) for 4 h (Ristova et al. 2016, Figure S16, Table S9). In fact, exposure to higher doses of KNO_3_ (10 mM) for 20 to 30 min significantly *repressed HHO5* gene expression in 2 other studies (Hu et al. 2009; Liu et al. 2022b, Figure S15b). Interestingly, in response to short-term temporal KNO_3_ treatments (Krouk et al. 2010b), *HHO5* expression was repressed at early time points but induced at later time points (Figure S15b). Moreover, transcriptomic studies of *nitrate reductase* (NR) double mutants revealed that nitrate accumulation in shoots (Monden et al. 2025) and in roots (Wang et al. 2004) resulted in strong and significant repression of *HHO5* expression (Figure S15b). Therefore, results from 5 independent datasets suggest that nitrate accumulation represses *HHO5* gene expression (Wang et al 2004; Hu et al. 2009; Krouk et al. 2010b; Liu et al. 2022b; Monden et al 2025;  Figure S15b, Fig. 8).

To mechanistically understand how nitrate represses *HHO5*, we searched through previous transcriptome studies of *nrt1.1* nitrate transceptor mutants (Hu et al. 2009; Bouguyon et al. 2015; Liu et al. 2022a). Our analysis revealed that *HHO5* was only differentially expressed in an *nrt1.1* mutant in one of these 3 datasets. Thus, there is not sufficient evidence to support that *HHO5* is directly downstream of NRT1.1 (Figure S17). However, in a study which uncovered a set of N-dose response genes that followed Michaelis-Menten (MM) kinetics, the MM response of *HHO5* was found to be under the control of the TF TGA1 (Swift et al. 2020, Figure S18). We mined this N-dose response dataset to discover that TGA1 represses expression of *HHO5* both *in planta* (Swift et al. 2020), and in the cell-based TARGET assay housed in the ConnecTF database (Brooks et al. 2021) (Figure S18a). Furthermore, mutating TGA1 *in planta* strongly increased the maximum rate of *HHO5* expression change in response to N-dose (Vmax, Swift et al. 2020) (Figure S18b). Altogether, nitrate repression of *HHO5* can be at least partially explained by TGA1, rather than direct input from NRT1.1.

As inorganic N signals could not explain the induction of *HHO5* expression in response to Total N treatments, we expanded our analyses to include organic N signaling datasets (Gutiérrez et al. 2008; Muñoz-Nortes et al. 2017; Goto et al. 2020). Indeed, we found that *HHO5* induction in response to Total N (60 mM N, NH_4_NO_3_ + KNO_3_) could be blocked by MSX, an inhibitor of glutamine synthetase (Gutiérrez et al. 2008) (Figure S15c). This finding suggests *HHO5* expression in response to N-treatments may depend on the conversion of inorganic to organic N. MSX inhibition of N-induced *HHO5* expression was restored by exogenous glutamate (Glu) treatment (Gutiérrez et al. 2008), suggesting that *HHO5* is induced by organic N signals (Figure S15c). The induction of *HHO5* expression by Glu treatments could represent a response to Glu, Glu-derived N-assimilation into organic N (Glu, Gln, Asp, Asn), or downstream organic N-derived products. Our analysis of data from an additional study shows that exogenous Glu treatments of Arabidopsis (Goto et al. 2020) can transiently induce *HHO5* gene expression relative to a urea treatment control (Figure S15c). Lastly, we found that *HHO5* expression was induced in glutamate synthase FD-GOGAT mutants (*glu1*), which over-accumulate glutamine (Muñoz-Nortes et al. 2017, Figure S15c). Therefore, across 3 independent studies, *HHO5* gene expression is induced by external treatment or internal accumulation of organic N (Glu/Gln) (Gutiérrez et al. 2008; Muñoz-Nortes et al. 2017; Goto et al. 2020).

### HHO5 orchestrates feedback regulation between inorganic and organic N signals

Overall, our meta-analysis supports a working model in which *HHO5* expression is repressed by inorganic N signals (nitrate) but induced by organic N signals (eg Glu/Gln) (Figure S15, Fig. 8). According to this model, high nitrate doses repress *HHO5* expression, while subsequent reduction and assimilation into organic N results in *HHO5* induction (Fig. 8). This working model for N-dose sensing can explain why *HHO5* is repressed by KNO_3_ in early time points but induced at later time points of a time-series analysis of KNO_3_ signaling (Krouk et al. 2010b, Figure S15b). We next modeled how HHO5 can act through 2 distinct pathways: as a repressor of nitrate-responsive genes and an inducer of organic-N-responsive genes.

First, we showed that HHO5 direct DNA binding is associated with repression of nitrate response genes, likely via direct repression of the nitrate transceptor *NRT1.1* (Figure 5d). This results in a negative feedback loop; nitrate accumulation represses *HHO5* expression, and HHO5 represses nitrate sensing and transport via *NRT1.1* (Figure S15, Fig. 8). Secondly, we found that HHO5 can also function as an inducer of organic N-response genes (Fig. 5a). Thus, in response to organic N signals, HHO5 also initiates a feedforward loop that induces glutamate-responsive organonitrogen and defense-related genes.

To better understand the effects of Glu signals on the HHO5 *in planta* N-dose GRN, we expanded our analysis of Glu-responsive genes to encompass the entire network (Fig. 7, Table S9, Figure S19). We found that the validated HHO5 GRN is significantly enriched for glutamate-response genes (*P* = 9.30×10^−10^) (Fig. 8). Interestingly, the network of *in planta* N-dose genes regulated by HHO5 is specifically enriched for genes whose expression is induced by glutamate (*P* = 1.72×10^−25^, Fig. 8, Figure S19) (Goto et al. 2020). Genes in the HHO5 N-dose GRN whose expression is induced by Glu treatments are enriched for GO terms related to nutrient level responses (7.48×10^−4^), amide transport (4.98×10^−2^), and systemic acquired resistance (1.70×10^−2^) (Fig. 7). Importantly, and in line with our working model, the validated HHO5 GRN is *not* enriched for genes that are repressed by glutamate treatments (*P* = 0.99, Fig. 7, Figure S19) (Goto et al. 2020). Glu treatment stimulates the expression of over half of the genes whose N-dose response *in planta* depends on HHO5. The validated HHO5 GRN is also significantly enriched in nitrate signal-specific responsive genes (*P* = 3.03×10^−23^), which were previously determined by a transcriptomic analysis of nitrate reductase null mutants (Figure S19) (Wang et al. 2004). Therefore HHO5 gene expression, and the expression of the HHO5 *in planta* target genes, encompasses both inorganic and organic N signals (Fig. 8, Figure S19).

**Figure 9:**
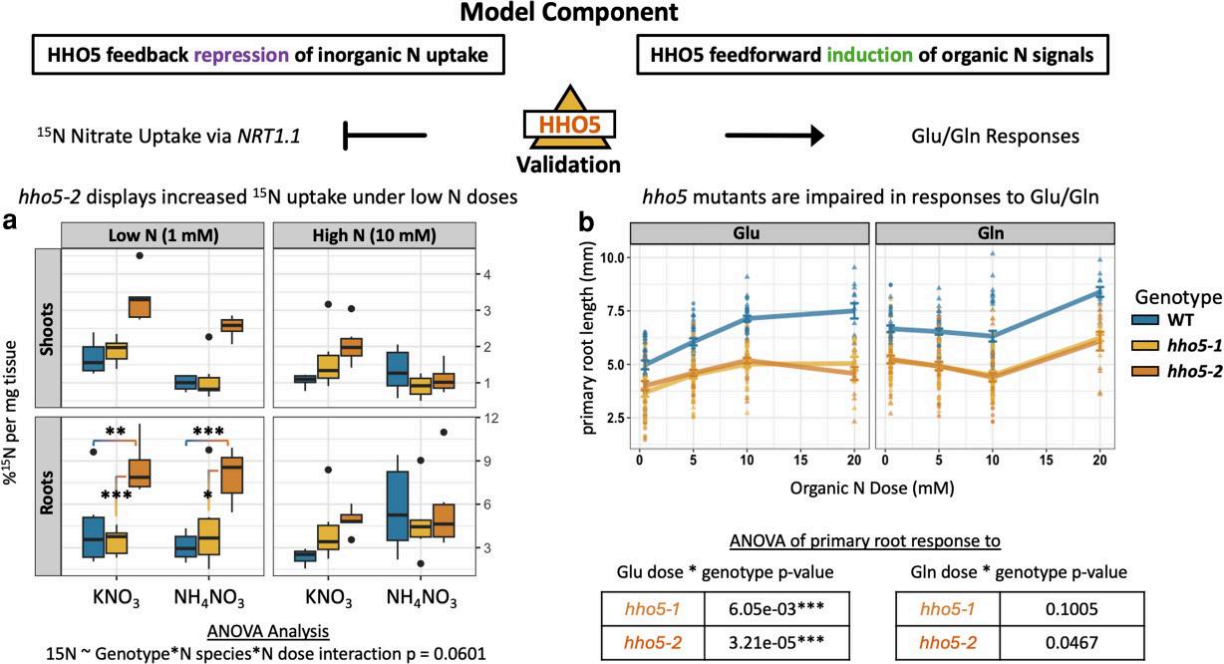
HHO5 represses inorganic N uptake, and induces responses to Glu *in planta* 2 main arms of the proposed HHO5 dual mode of action over inorganic and organic N signaling are summarized above their respective phenotypic validation datasets. a) ^15^N uptake of KNO_3_ or NH_4_NO_3_ in low (1 mM) or high (10 mM) N conditions, in WT and both *hho5* T-DNA mutants. *hho5-2* is the stronger mutant allele, see Figure S3a). Data from shoots are shown above data from roots. Significant differences reflect adjusted *P*-values from ANOVA using the model: 15N∼Genotype*N-species*N-dose*Tissue with a TukeyHSD test. Significance (*P* < 0.05) is only shown for comparisons within the 4 plot quadrants. b) Primary root length in response to varying doses of Glu (left) or Gln (right). ANOVA revealed significant Glu-dose dependent genotype effects on primary root lengths. Error bars represent standard error of the mean.

To validate our working model of HHO5 dual activity over N-dose signaling, we completed *in planta* phenotype experiments to address both of the proposed HHO5 centric feedback loops (Fig. 8). We first completed ^15^N uptake assays, which revealed that the *hho5-2* mutant displays dose-dependent increases in ^15^N uptake. This effect was significant in roots grown in low N doses, and trended in the same direction in shoots (Fig. 9a). In contrast, this effect was attenuated under higher N conditions, suggesting that HHO5-mediated repression is most relevant under limiting N availability. The *hho5-2* mutant is a stronger mutant than *hho5-1* (Figure S3a, Fig. 4a), explaining why the weaker *hho5-1* mutant may still display functional repression of inorganic N uptake. The de-repression of ^15^N uptake in *hho5-2* was N dose-dependent, as described by a significant genotype * N-dose interaction effect in ANOVA (*P* = 3.41e^−04^). These results support our model of dose-sensitive repression of inorganic N uptake by HHO5 (Figs. 8 and 9).

In addition to HHO5 repression of nitrate uptake in low N conditions, we propose that HHO5 also promotes organic N signals in response to increasing organic N dose (Fig. 8). Our previous *hho5* mutant phenotype experiments were grown on KNO_3_, NH_4_NO_3_, or total N treatments, which in long-term growth should initiate both inorganic and organic N responses. To test if *hho5* mutants are impaired in their responses specifically to organic N signals, we grew WT and both *hho5* mutants on multiple doses of either L-Glutamate (Glu) or L-Glutamine (Gln) and measured primary root growth (Fig. 9b). While both *hho5* mutants were impaired in responses to both Glu and Gln, the mutants only displayed dose-dependent responses to Glu. The significant N-dose* Genotype ANOVA *P*-values indicate that dampened primary root growth in both *hho5* mutants intensifies with increasing Glu. HHO5 is thus required for N-dose dependent primary root responses to glutamate (Fig. 9).

In total, we found that HHO5 expression is induced by organic N signals and repressed by inorganic N signals. In response to organic N, HHO5 regulates a GRN that includes both inorganic and organic N responsive genes. When combined with its dual regulatory mode-of-action, we show HHO5 acts as a repressor of nitrate uptake and inducer of organic-N responses at the gene expression and phenotype levels. Based on the phloem-specific expression of *HHO5* and some of its N-dose response target genes, we hypothesize that *HHO5* is an orchestrator of feedback repression of inorganic N signaling when high levels of organic N are being transported in the phloem (Lam et al. 1995) (Fig. 8).

## Discussion

In this study, we highlight the role that HHO5 plays in dose-dependent inorganic and organic N signal regulation. HHO5 had been identified as N-responsive in previous datasets but had not yet been functionally characterized in the context of N dose signaling. We found multiple ways in which HHO5 is diverged from other members of the HHO TF family. Despite sharing conserved *MYB* DNA-binding domains and hydrophobic globular domains (HGD), *HHO5* displays structural differences from the other HHO TFs in regard to their ERF-associated *Amphiphilic Repressor* (*EAR)*-like domains (Li et al. 2021). The EAR-like domain confers target gene repression and facilitates protein–protein interactions with EAR-like domains and non-EAR-like protein domains (Tiwari et al. 2004; Kagale et al. 2010). *HHO5* is unique in carrying 2 EAR-like domains, located at each of the 5′ and 3′ ends (Li et al. 2021). By contrast, *HRS1*, *HHO2*, and *HHO3* each contain a single EAR-like domain, while *HHO1*, *HHO4*, and *HHO6* do not contain any EAR-like domains (Li et al. 2021). We postulate that the additional EAR-like motif at the 3′ end of *HHO5* may enhance HHO5's interaction potential with partner TFs, suggesting a possible structural basis for the functional divergence of HHO5 from the *NIGT1* TFs of the HHO family.

As a family, the well-studied HHO TFs (*NIGT1s*) preferentially function as direct transcriptional repressors (Figure S8), which is consistent with their EAR-like domains (Kiba et al. 2018; Li et al. 2021; Safi et al. 2021). In this context, direct repression refers to direct contact between HHO TFs and DNA and corresponding target gene repression (Figure S8). The association between direct DNA binding and gene repression holds for HHO5. This engenders the question; How does HHO5 induce target gene expression? We propose that gene induction by HHO5 can be accomplished by 2 possible (and not mutually exclusive) explanations. Our first hypothesis is that HHO5 directly regulates TF2s, which then initiate a cascade of transcriptional N-dose signals. Our second hypothesis is that HHO5 induces target genes by indirectly binding DNA via a partner TF.

Our data provide support for both hypotheses. In support of hypothesis 1, we found that HHO5 regulates multiple downstream TFs (TF2s) that are well established inducers of gene expression (eg *bZIP1, WRKY33, WRKY75*) (Birkenbihl et al. 2012; Para et al. 2014; Zhang et al. 2018). Thus HHO5 directly regulates TF2s that can induce target gene expression after organic N-dose stimulus via HHO5 (Fig. 7). Our Network Walking analysis uncovered a network path downstream of HHO5. We found that experimentally validated activity of HHO5 and the HHO5-TF2s accounted for almost 70% of the HHO5 regulated N-dose responsive genes observed *in planta* (Fig. 7). These findings position HHO5 as a key regulator of N signaling through hierarchical transcriptional control of HHO5-TF2s.

However, this TF2-based hypothesis of HHO5 target gene induction fails to explain how HHO5 itself could directly induce target genes. To address this, we propose a second hypothesis, which is that HHO5 indirect DNA binding via partner TFs leads to HHO5 target gene induction. The presence of the extra EAR-like domain in *HHO5* that facilitates protein-protein interactions complements this hypothesis well. Toward identifying putative HHO5 partner TFs, we found that the genes directly induced (but not directly bound) by HHO5 in cells are highly enriched for the *WRKY* TF family cis motif (Fig. 5b). Thus, *WRKY* TFs may aid HHO5 in inducing the expression of N-dose responsive genes. This hypothesis is supported by the fact that many WRKY TFs contain EAR domains (Kagale et al. 2011). Therefore, WRKY TFs are structurally compatible partners for physical interaction with HHO5, and are also well characterized master regulators of plant immunity (Liu et al. 2015; Javed et al. 2023), which was functionally enriched among the HHO5 induced genes (Fig. 5).

From our analysis, we identify WRKY21 as a potential partner TF mediating HHO5-dependent gene induction. WRKY21 is spatially and temporally co-expressed with HHO5 (Fig. 6). Importantly, DAP-seq data indicates that WRKY21, but not HHO5, directly binds the promoters of HHO5-induced genes, supporting a model in which WRKY21 facilitates HHO5 interaction with its induced targets (Fig. 6c). Our new DoubleTARGET assay further supports this interaction, as co-expression of *HHO5* and *WRKY21* in root protoplasts results in enhanced induction of a subset of HHO5 target genes relative to either TF alone. These synergistically induced genes are enriched in defense-related functions (Fig. 6e). Together, these results support a model in which WRKY TFs switch HHO5 from a repressor into a gene inducer, providing a mechanistic basis for HHO5 transcriptional dual activity.

Another way in which HHO5 is diverged from the *NIGT1* TFs is with its distinct phloem expression, detected in isolated root cells and also by vascular-specific expression of an HHO5-GUS fusion in shoots and roots of whole plants (Fig. 2). The phloem-specific expression of *HHO5*, and some of its targets in the GRN (Figure S14), positions it to mediate responses to systemic levels of N resources. While the NIGT1 TFs and HHO5 share some protein domains, *HHO5* is expressed specifically in phloem, meaning HHO5 has access to different partner TFs and chromatin contexts. In summary, direct DNA binding and repression of nitrate response genes is conserved across the HHO TFs, including HHO5. However, the biological context in which the HHO TFs are placed leads to different functional outcomes on plant N-signaling. We also found that HHO5 uniquely induces genes related to organic N responses and defense, which were not regulated by HHO2 and HHO3 (Figure S7). This dual regulatory activity of HHO5 in phloem cells positions HHO5 as a key integrator of inorganic and organic N signaling.

The specific expression of HHO5 in phloem cells (Fig. 2) may explain why perturbing *HHO5* has a uniquely strong effect on N-dose dependent gene expression and phenotypes (Fig. 3). The specific expression of HHO5 in phloem pole cells is intriguing as these cells are involved in long-distance transport of sugar and other metabolites (Liu et al. 2022a). Phloem cells are important for transporting nitrogenous amino acids to developing tissues (Lam et al. 1995; Okumoto et al. 2004), which explains how phloem-localized HHO5 can sit at the interface of both inorganic and organic N signaling. Accordingly, we found that HHO5 regulates genes related to nutrient starvation and nitrate import that are expressed highly in phloem cells (Figure S14). That HHO5 induces organonitrogen genes and is localized in phloem could also explain the decreases in seed N content in the *hho5-1* mutant (Fig. 4). Thus, we propose that HHO5 may be an important phloem-specific regulator of systemic organic N signaling both at the gene expression and phenotype levels.

Our meta-analysis and model suggests that induction of HHO5 expression by organic N represses nitrate uptake (Fig. 8). This HHO5-centric negative feedback loop is a possible mechanism by which organic N signals can repress energy intensive reactions involving inorganic N uptake/reduction/assimilation to conserve energy under high N-doses (N-satiety) (Figs. 5 and 8).

In our study, we found *hho5* T-DNA mutants to be smaller than WT plants in an N-dose dependent manner (Fig. 4a). The decreased growth rates in *hho5* mutants could be reflective of our finding that HHO5 induces organic N related genes both in cells and *in planta* (Fig. 5, Figure S7). In accordance with this, we found that around half of the validated HHO5 N-dose GRN was induced by exogenous glutamate response genes (Goto et al. 2020) and was associated with growth related terms such as amide transport and response to nutrient levels (Fig. 8).

As glutamate is a metabolite associated with defense, the organic N-mediated HHO5 feedback loop could also contribute to tradeoffs between growth and defense, which were terms enriched in the organic-N arm of the HHO5 GRN (Fig. 7). In response to a pathogen, induction of *HHO5* expression by glutamate could lead to induction of defense-related genes in our HHO5 GRN *(CHI, LYK5*, Goto et al. 2020), while repressing nitrate uptake and assimilation to conserve energy (Fig. 5, Figure S7). This model is further supported by a recent study (Hernández-Coronado et al. 2022) showing glutamate receptors, including the phloem-expressed HHO5 target gene *GLR1.2* (Figure S20), sit at the interface of growth and defense tradeoffs. Another HHO5 target gene, *GLR1.1* was shown to regulate N/Carbon metabolism balance (Kang et al. 2003). Our data support that organic-N signaling by HHO5 directly induces 5 N-dose responsive glutamate receptors both in cells and *in planta* (Figure S7).

Importantly, phenotypic analyses *in planta* are consistent with this dual regulatory model. ^15^N influx assays revealed that *hho5-2* mutants display increased inorganic N uptake, supporting a role for HHO5 as a negative regulator of nitrate and ammonium acquisition (Fig. 9). This effect was dependent on N dose, as HHO5-mediated repression of inorganic N uptake is the strongest under limiting N availability. Complementary to this, root growth assays showed that *hho5* mutants are impaired in their response to exogenous organic N, particularly glutamate, exhibiting decreased primary root growth across Glu gradients. Together, these findings provide empirical evidence that HHO5 represses inorganic N uptake while promoting responses to organic N, reinforcing its role as a dual regulator of N-dose signaling.

There are thus multiple lines of evidence suggesting HHO5 and possibly defense-associated WRKY Partner TFs, may integrate organic N signals to balance growth and defense. Further study of this growth/defense tradeoff could have important agricultural implications, especially considering that HHO5 is a key hub of a highly conserved N-response network conserved between Arabidopsis and rice (Obertello et al. 2015). Nitrate reduction requires a considerable amount of energy. Thus, under organic N satiety or pathogen attack, HHO5 is possibly dampening nitrate uptake and assimilation as a way of signaling that the plants can shunt their energy toward non-N-related processes. In the context of N-dose, nitrate accumulation represses *HHO5*. Nitrate repression of *HHO5* in turn activates nitrate uptake and assimilation (repression of a repressor engenders activation). Once the nitrate is assimilated, the resulting organic N induces *HHO5* expression to promote organic N metabolism. However, as a dual inducer/repressor TF, HHO5 would also repress further uptake of nitrate under organic N satiety. This mechanism would also be beneficial during pathogen attack. In this case, defense-related accumulation of Glu could induce HHO5, which in turn represses energy intensive nitrate uptake as a means of energy conservation. In total, we propose HHO5 sits at the center of a feedback mechanism between organic and inorganic N signaling, which could in turn influence plant growth and defense in specific N-dose conditions.

## Supplementary Material

koag201_Supplementary_Data

## Data Availability

Both *hho5* T-DNA mutants are available for ordering from the Arabidopsis Biological Resource Center (ABRC) under the following accessions: *hho5-1* (SAIL_806_F06), *hho5-2* (SALK_077802) (O’Malley et al. 2015). The *in planta* transcriptome dataset from this study (Figure S1) was made available at the Gene Expression Omnibus (GEO) database under accession number PRJNA512225. Gene expression counts were made available as Table S10. DoubleTARGET raw RNA-seq reads and the associated count matrix were made available in GEO as GSE329773. Plasmids used in DoubleTARGET are available at the VIB stock center under accessions 5_68 (GR:EV, RFP) and 10_69 (GR:EV, GFP).
