# Supplementary material for: HHO5 orchestrates dose-dependent feedback regulation of organic versus inorganic nitrogen signaling in Arabidopsis
Source: Plant Cell. 2026 Jul 30;38(7):koag201. doi: 10.1093/plcell/koag201 (PMC13421923; doi:10.1093/plcell/koag201)
Supplement: koag201_Supplementary_Data [file koag201_supplementary_data.zip › 260519_HHO5_Supplemental_Figures.pdf]

Supplemental Materials

Supplementary Table S1: 4,464 N-dose DEGs in Col-0 roots with significance statistics from DESeq2 and cluster identity corresponding to supplementary Fig. 2 clusters.

Supplementary Table S2: Complete list of enriched GO terms in the N-dose activated or N-dose repressed genes shown in Fig. 1A.

Supplementary Table S3: 2,894 Genotype DEGs, which are genes differentially expressed in both hho5 T-DNA mutant roots compared to WT (Col-0), with DESEq2 statistics.

Supplementary Table S4: 828 HHO5-regulated N-dose genes in planta (N-dose & Genotype DEGs, and cluster identity from supplementary Fig. 4).

Supplementary Table S5: 1,524 HHO5-direct target genes in shoots from the cell-based TARGET assay (Varala et al., 2018).

Supplementary Table S6: Complete list of enriched GO terms in the HHO5-directly-activated or HHO5-directly-repressed genes from the TARGET assay in Fig. 4A.

Supplementary Table S7: HHO5 DAP-seq and ampDAP-seq target genes (within 500-BP of peaks) from the ConnecTF.org database (Brooks et al., 2021).

Supplementary Table S8: Network Walking GRN of nodes and edges linking HHO5 to 567 HHO5-regulated N-dose genes *in planta.*

Supplementary Table S9: Results from the Meta-Analysis intersection of the Network Walking GRN (Supplementary Table S8) with N-related genomic datasets.

Supplementary Table S10: *in planta* transcriptome experiment raw gene expression counts (corresponding to Supplementary Fig. 1).

Supplementary Table S11: Table of primers used in this study.

Supplementary Fig. 1: Transcriptome-wide WT and *hho5* mutant-dependent responses to N-dose in Arabidopsis roots are revealed by PCA.

Supplementary Fig. 2: Total N treatments perturb thousands of genes relative to a KCl treatment control.

Supplementary Fig. 3: HHO5 regulates thousands of genes *in planta.*

Supplementary Fig. 4: HHO5 regulates 828 N-dose response genes *in planta.*

Supplementary Fig. 5: *hho5* mutants show significant N-dose dependent growth changes regardless of which N-species are in growth media.

Supplementary Fig. 6: *hho5* mutants display significant defects in N-dose dependent growth of the primary root.

Supplementary Fig. 7: HHO5 regulates organonitrogen-related genes.

Supplementary Fig. 8: HHO TFs preferentially bind the HHO5-repressed genes.

Supplementary Fig. 9: A subset of WRKY TFs respond to N-dose signals and are highly expressed in phloem cells.

Supplementary Fig. 10: DoubleTARGET co-overespression HHO5 and WRKY21 in Arabidopsis root protoplasts.

Supplementary Fig. 11: DoubleTARGET co-perturbs HHO5 and WRKY21 in Arabidopsis root protoplasts.

Supplementary Fig. 12: HHO5 directly regulates 145 N-dose genes in cells and *in planta*.

Supplementary Fig. 13: HHO5 initiates a signaling cascade of 53 N-dose responsive TFs.

Supplementary Fig. 14: HHO5 regulates 64 N-dose genes in phloem that are enriched for functions related to “regulation of root development”.

Supplementary Fig. 15: *HHO5* is repressed by nitrate but induced by organic N.

Supplementary Fig. 16: *HHO5* does not respond to four hour, low dose treatments of nitrate or ammonium.

Supplementary Fig. 17: *HHO5* is not consistently misregulated in *nrt1.1* mutant studies.

Supplementary Fig. 18: TGA1 represses the Michaelis-Menten mediated N-dose response of HHO5.

Supplementary Fig. 19: The HHO5 GRN is significantly enriched for nitrate-specific and glutamate induction responses.

Supplementary Fig. 20: *HHO5*, *bZIP1*, *TGA1*, and *GLR1.2* are expressed in phloem.

Supplementary Fig. 21: Confirmation of two null HHO5 T-DNA mutants.

Supplementary Figures

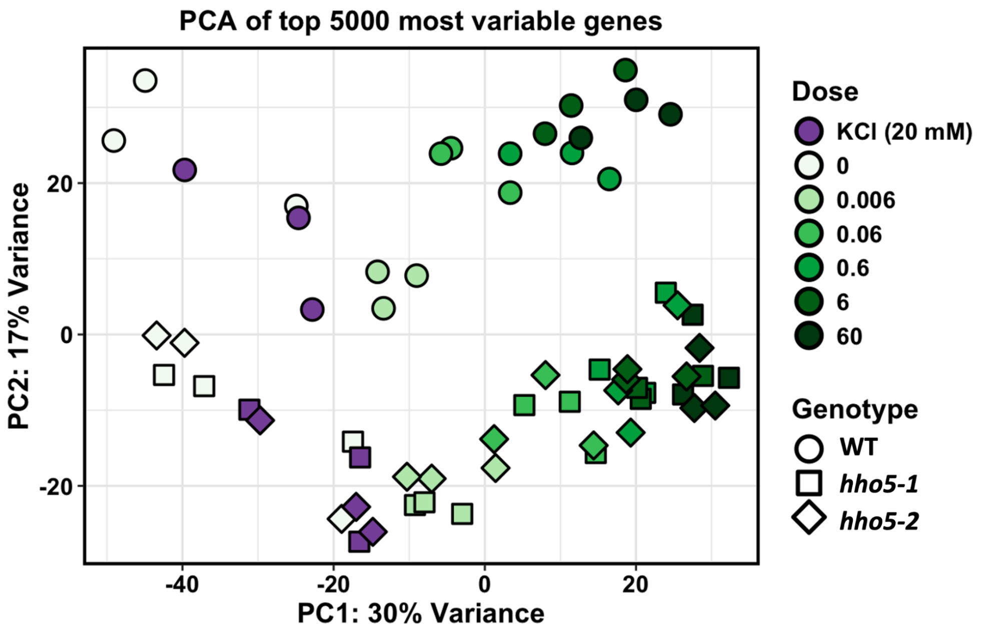

Supplementary Figure 1: Transcriptome-wide WT and *hho5* mutant-dependent responses to N-dose in Arabidopsis roots are revealed by PCA
Principal Component Analysis (PCA) of the top 5000 most variable genes in Arabidopsis roots, showing variation across genotypes (shape) and nutrient treatments (colors). Supports Fig 1.

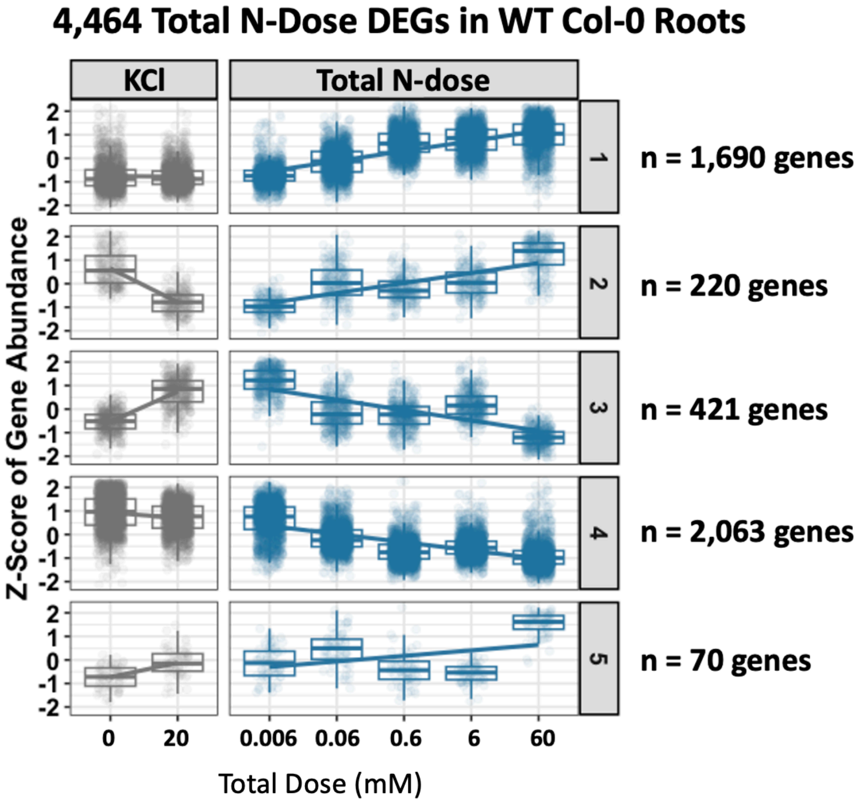

Supplementary Fig. 2: Total N treatments perturb thousands of genes relative to a KCl treatment control
Z-score scaled gene expression of the 4,464 N-dose DEGs in response to N-dose and in the KCl controls (labeled C for Control, shown in grey as 0 and 20 mM KCl). Supports Fig 1.

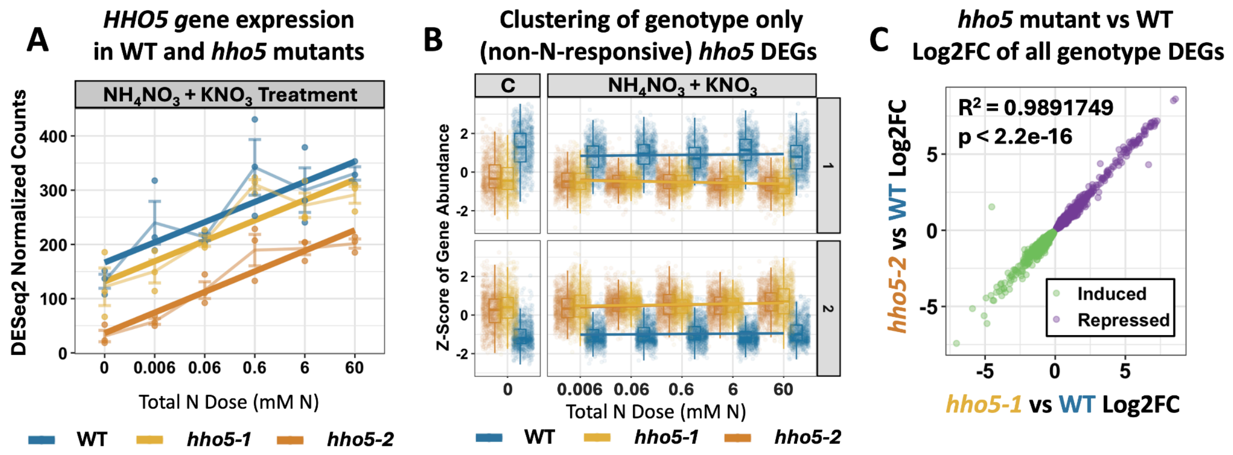

Supplementary Figure 3: HHO5 regulates thousands of genes *in planta*

A) HHO5 gene expression across genotypes and N-doses. B) Expression profiles of genes induced (top) and repressed (bottom) by HHO5, but not regulated by N-dose. C) Correlation of Log2FC between hho5-1 and hho5-2 mutants for the 2,894 genes regulated by HHO5 in planta. Supports Fig 3.

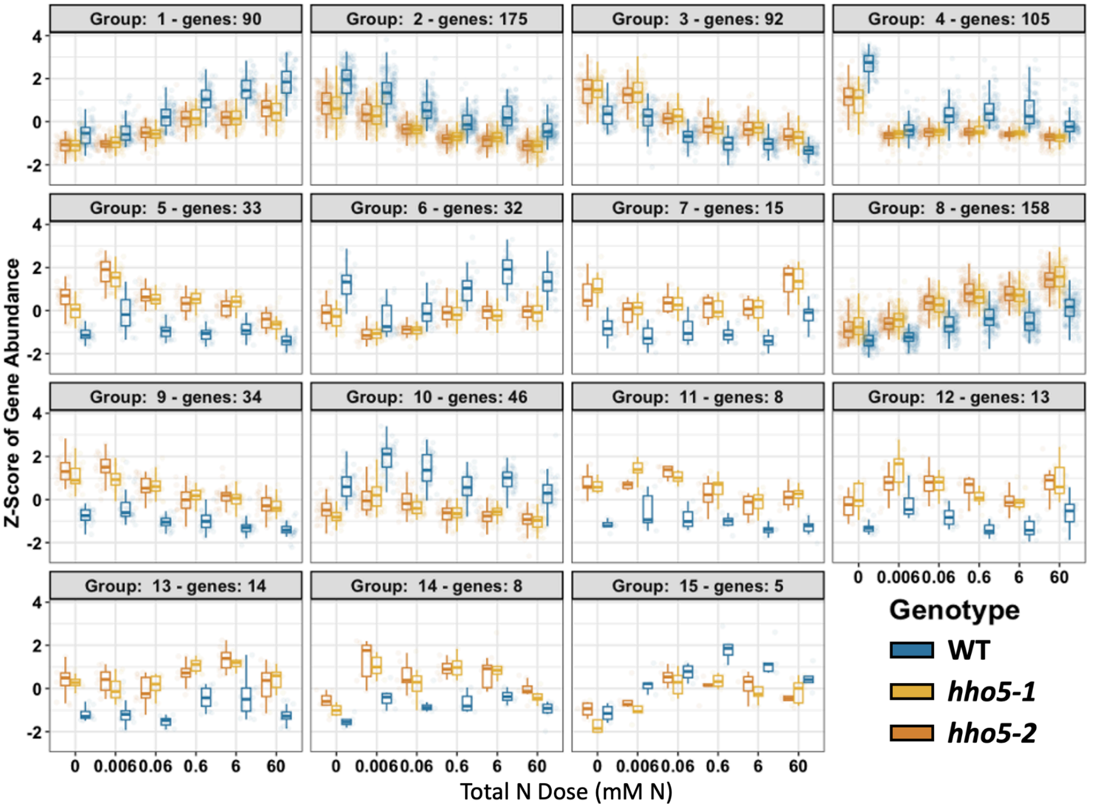

Supplementary Figure 4: HHO5 regulates 828 N-dose response genes *in planta*

15 gene expression clusters of z-score scaled gene expression across N-doses in WT and two hho5 T-DNA mutants. N-dose represents Total N dose as described in Fig. 1. Supports Fig 3.

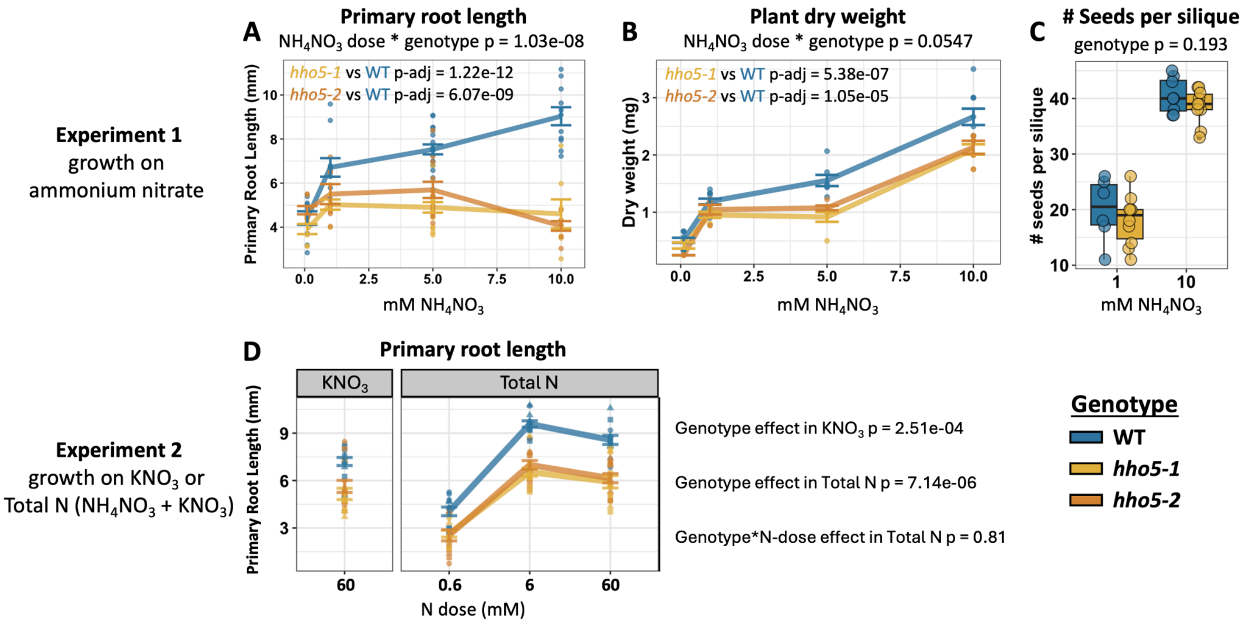

Supplementary Figure 5: *hho5* mutants show significant N-dose dependent growth changes regardless of which N-species are in growth media

*A)Primary root length and (B) plant dry weight measured across NH_4NO_3 doses in WT and hho5 mutants, where an ANOVA was performed to test for a significant genotype*N-dose interaction effect. C) Number of seeds per silique. D) Primary root length on plants growing on 60 mM KNO_3 (the total amount of N present in MS media), and in three different of total N doses (mM).Supports Fig 4.*

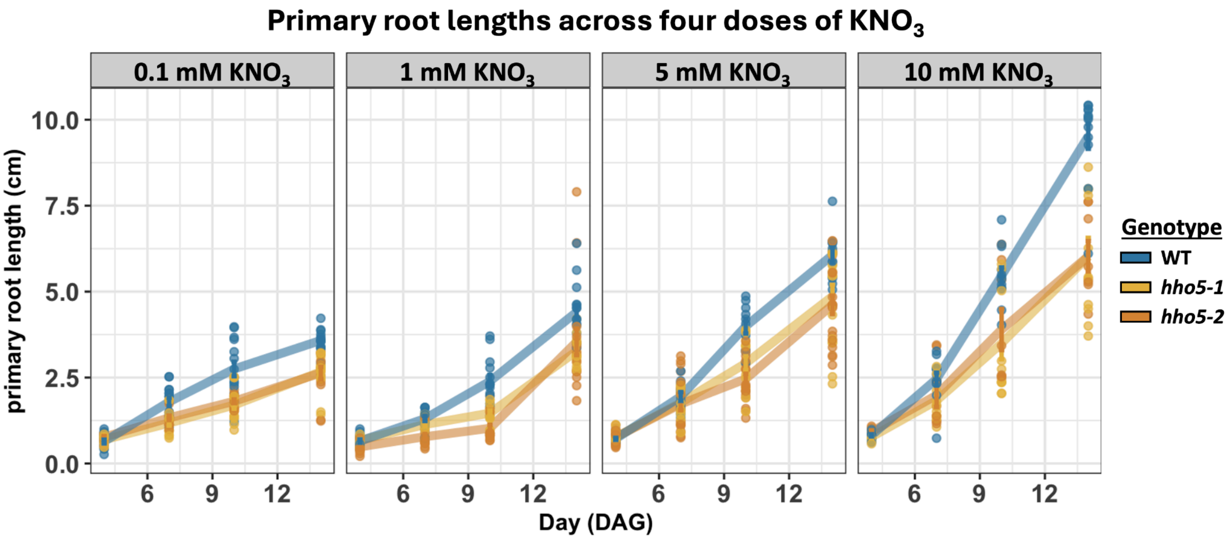

Supplementary Figure 6: *hho5* mutants display significant defects in N-dose dependent growth of the primary root

Plots of primary root lengths across 4 doses of KNO_3 (0.1, 1, 5, 10 mM) across 4 time points. Supports Fig 4.

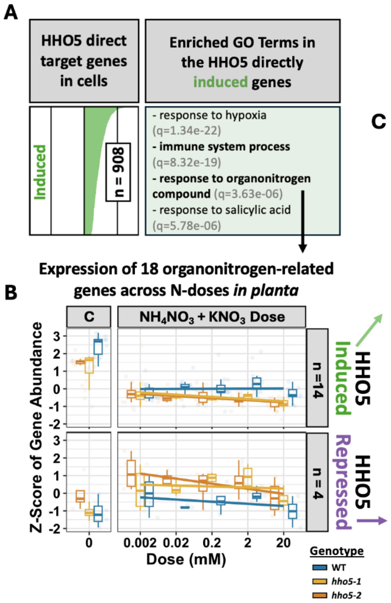

14 organonitrogen-related genes are

A) Directly **induced** by HHO5 in the cell-based TARGET system.
B) **Induced** by HHO5 across N-doses *in planta* (expressed lower in *hho5* T-DNA mutants).
C) Not regulated, or **repressed**, by the NIGT1 TFs with available TARGET data (*HHO2, HHO3*).
D) Most are significantly **induced by glutamate** treatments (based on Goto et al 2020).

C **Table of TARGET direct TF-gene regulation data**

14 organonitrogen genes directly induced by HHO5 in TARGET and *in planta*

HHO5 target genes		Focus TF: *HHO5*		HHO5 Partner TF?: *WRKY33*		HHO5 Paralog: *HHO6*		NIGT1: *HHO2*		NIGT1: *HHO3*	
Gene	AT ID	p-value	Log2FC	p-value	Log2FC	p-value	Log2FC	p-value	Log2FC	p-value	Log2FC
CERK1 (RLK)	AT3G21630	6.29E-04	5.61E-01			1.82E-02	-4.12E-01				
CHI	AT2G43570	6.75E-14	1.04E+00	2.01E-08	1.95E+00	1.53E-02	4.01E-01				
GLR1.2 (GLR)	AT5G48400	2.53E-12	9.08E-01	3.74E-06	5.88E-01			3.53E-02	-2.51E-01		
GLR1.3 (GLR)	AT5G48410	2.78E-10	9.16E-01	1.74E-06	1.07E+00	1.90E-03	4.98E-01				
GLR2.5 (GLR)	AT5G11210	4.06E-04	7.17E-01	2.99E-04	4.94E-01						
GLR2.8 (GLR)	AT2G29110	7.59E-13	1.54E+00			9.60E-03	-6.55E-01				
GLR2.9 (GLR)	AT2G29100	4.12E-02	1.01E+00					2.22E-02	8.35E-01		
LYK4 (RLK)	AT2G23770	7.29E-04	1.07E+00			9.55E-03	8.43E-01				
LYK5 (RLK)	AT2G33580	1.21E-03	6.65E-01			7.39E-05	7.60E-01	3.67E-02	-3.19E-01		
MKP1	AT3G55270	1.47E-03	5.65E-01								
PEN3 (ABC)	AT1G59870	1.13E-09	1.03E+00	2.34E-02	4.47E-01	1.94E-03	5.78E-01	7.82E-03	-4.11E-01		
RHL41 (TF)	AT5G59820	1.90E-02	4.58E-01								
ZAT11 (TF)	AT2G37430	8.93E-07	9.03E-01	4.24E-03	5.38E-01						
ZAT18 (TF)	AT3G53600	1.20E-14	1.47E+00	1.25E-02	5.37E-01						
**4 organonitrogen genes induced by HHO5 in TARGET, but repressed by HHO5 *in planta***											
ABCG37 (ABC)	AT3G53480	3.52E-25	1.28E+00			4.92E-30	1.38E+00	3.67E-28	1.62E+00	3.17E-12	1.09E+00
GLR1.1 (GLR)	AT3G04110	2.89E-02	7.74E-01	8.50E-05	1.91E+00						
IOS1 (RKL)	AT1G51800	6.22E-05	8.22E-01								
ZAT17 (TF)	AT2G28710	1.76E-36	2.36E+00			8.17E-21	1.77E+00				

Gene Label (Goto et al 2020)
Glu-Induced
Glu-Repressed

blank = not regulated

Supplementary Figure 7: HHO5 regulates organonitrogen-related genes

A) Genes induced by HHO5 in TARGET are enriched for organonitrogen-related functional annotations. B) Expression of the 18 organonitrogen genes from A across N-doses in planta. C) Mining TARGET for HHO2, HHO3, HHO6, and WRKY33 (from ConnecTF, Brooks et al. 2021) revealed these organonitrogen genes are not regulated, or repressed by the NIGT1 TFs. Genes significantly induced by glutamate treatments are highlighted in orange (Goto et al. 2020, FDR<0.01). Supports Fig 5.

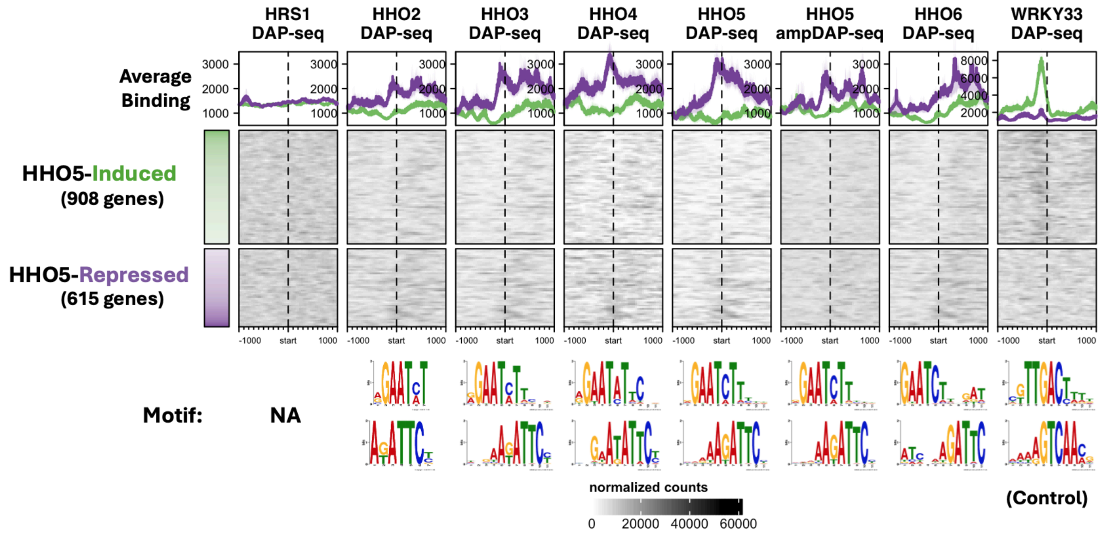

Supplementary Figure 8: HHO TFs preferentially bind the HHO5-repressed genes

Analysis of HHO5 DAP-seq and ampDAP-seq binding (O'Malley and Huang et al. 2016) over the HHO5 direct target genes from TARGET assay (Varala et al. 2018). This is plotted +/- 1 kb from the gene transcription start sites. DEGs are ordered by Log2FC. Lack of binding in HRS1 is likely due to its very low number of peaks (883), and a low fraction of reads under the peaks (~3%). Supports Fig 5.

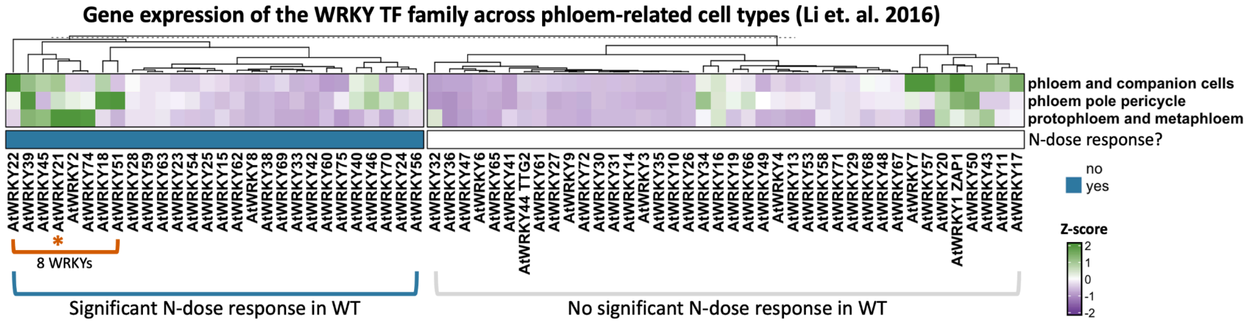

Supplementary Figure 9: A subset of WRKY TFs respond to N-dose signals and are highly expressed in phloem cells

Heatmap showing z-score scaled gene expression of 68/72 WRKYs that are expressed in the Li et al. 2016 root cell type atlas. WRKYs are grouped into TFs that display a significant N-dose response in our WT N-dose dataset (Figure 1, Supplemental Figure 1), versus the WRKYs that do not display a N-dose related gene expression response. WRKY21 is highlighted with an orange star as a putative partner TF. Supports Fig 6.

Collection of GFP/RFP double positive cells by FACS

A

Sample	Construct	Marker	# Collected Cells
1	Empty Vector	GFP+ Only	11,470
2			9,184
3			15,756
4			17,612
5	HHO5	GFP+ Only	19,172
6			15,416
7			17,853
8			16,059
9	WRKY21	RFP+ Only	18,110
10			16,623
11			15,923
12			15,252
13	HHO5 + WRKY21	GFP+/RFP+ Double Positive Only	11,035
14			13,833
15			15,268
16			15,338

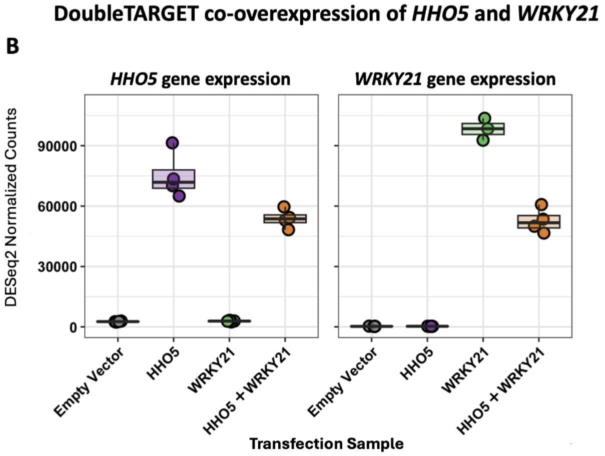

Supplementary Figure 10: DoubleTARGET co-overexpression of *HHO5* and *WRKY21* in Arabidopsis root protoplasts

A) Number of successfully transfected or co-transfected cells collected by FACS sorting on a BD DiscoverS8. B) Gene expression of HHO5 or WRKY21 across different transfection samples. Supports Fig 6.

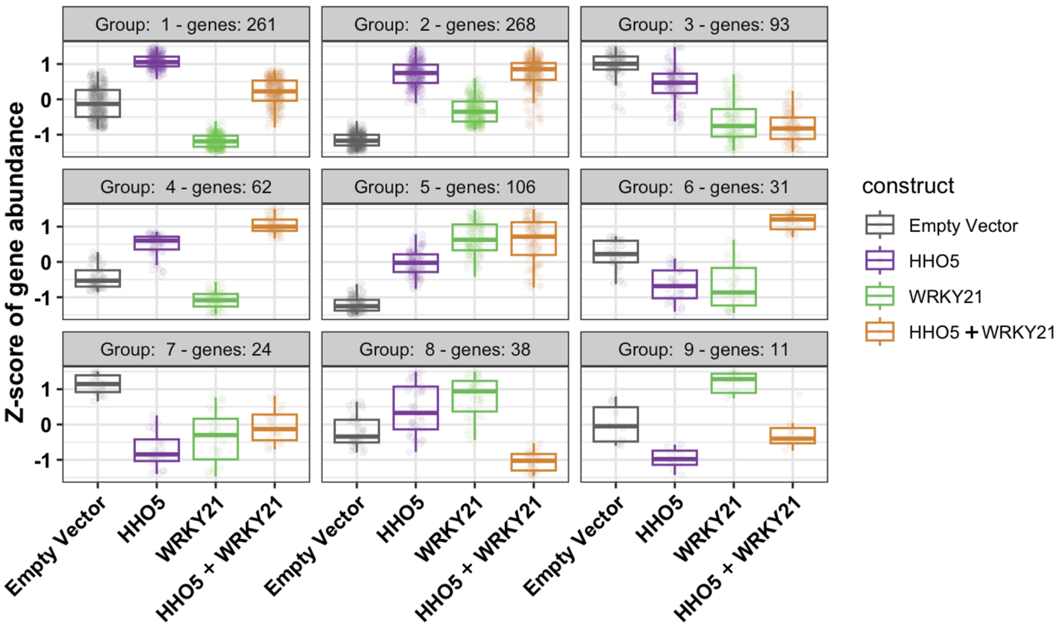

Supplementary Figure 11: DoubleTARGET co-perturbs HHO5 and WRKY21 in Arabidopsis root protoplasts

A) Z-score scaled gene expression of 908 validated HHO5 induced target genes across the HHO5/WRKY21 DoubleTARGET experiment (Varala et al. 2018, Supplementary table S5, Figure 5). Supports Fig 6.

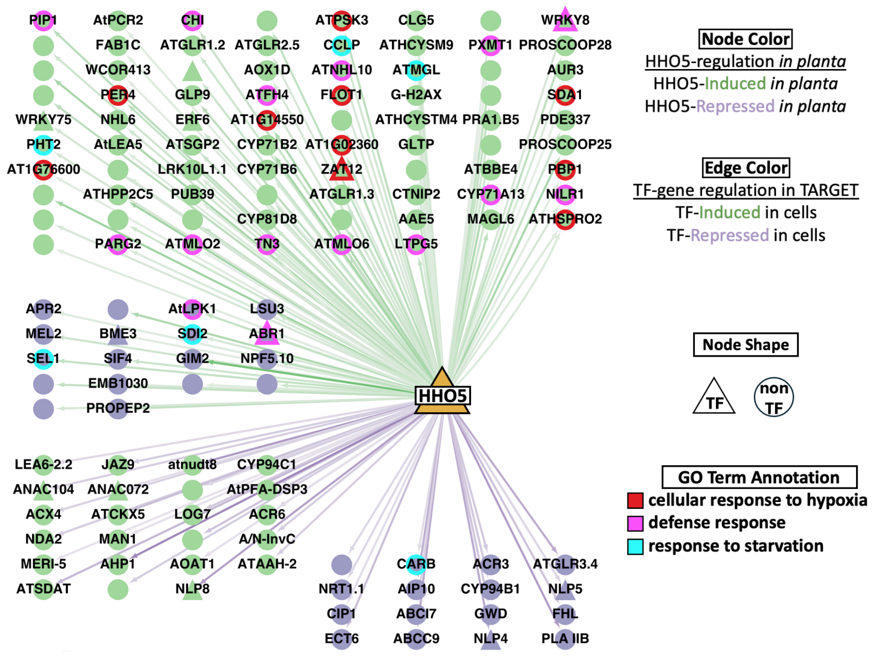

Supplementary Figure 12: HHO5 directly regulates 145 N-dose genes in cells and *in planta*

A subset of the Network Walking GRN highlights 145 N-dose genes downstream of HHO5, most of which (101/135) are directly induced by HHO5 (in the TARGET system, green edges). Supports Fig 7.

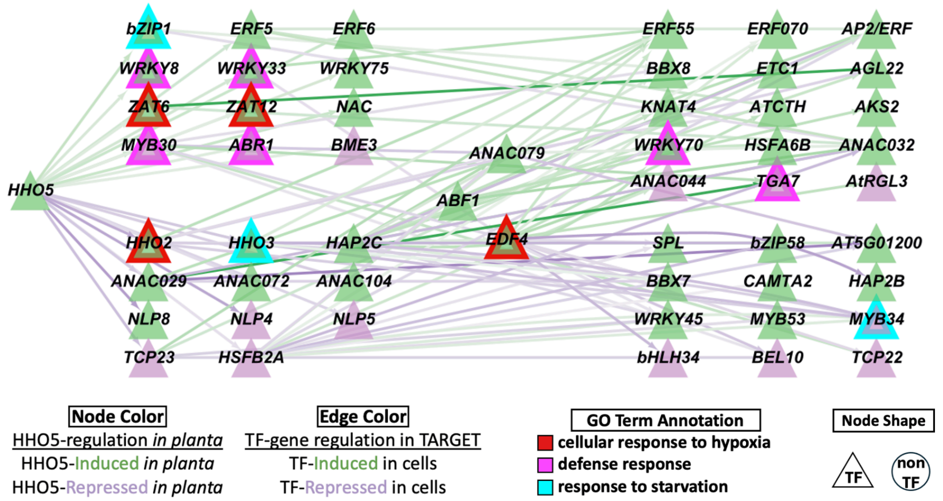

Supplementary Figure 13: HHO5 initiates a signaling cascade of 53 N-dose responsive TFs

Network showing all regulatory edges between HHO5 and the 53 validated N-dose responsive TFs downstream of HHO5 using TARGET data. 40 of the 53 are induced by HHO5 in planta. Line color denotes intensity of Log2FC in TARGET. Supports Fig 7.

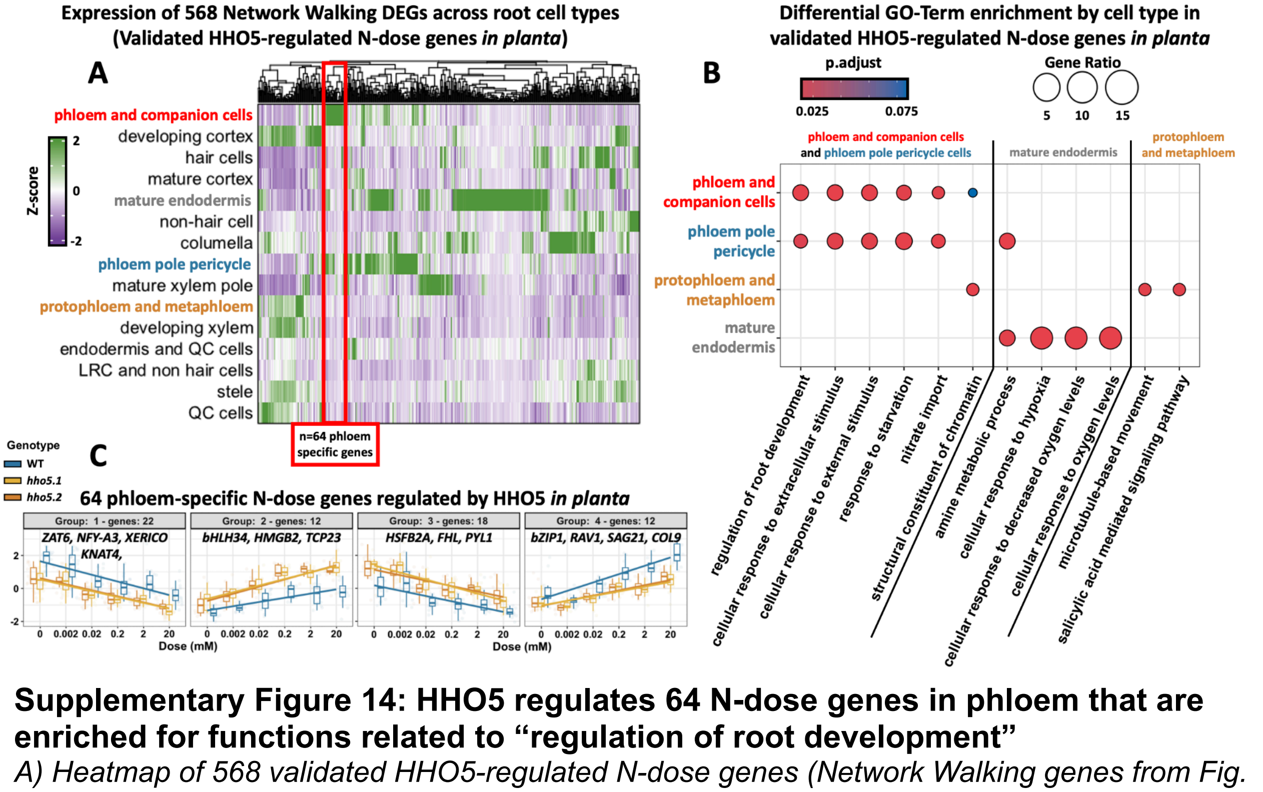

Supplementary Figure 14: HHO5 regulates 64 N-dose genes in phloem that are enriched for functions related to “regulation of root development”

A) Heatmap of 568 validated HHO5-regulated N-dose genes (Network Walking genes from Fig. 7) across the root cell type atlas (Li et al. 2016). Genes highly expressed in phloem and companion cells (Z-score >1) were selected for gene ontology enrichment analysis. B) GO-Term enrichment across cell types, with mature endodermis as a control. Supports Fig 7.

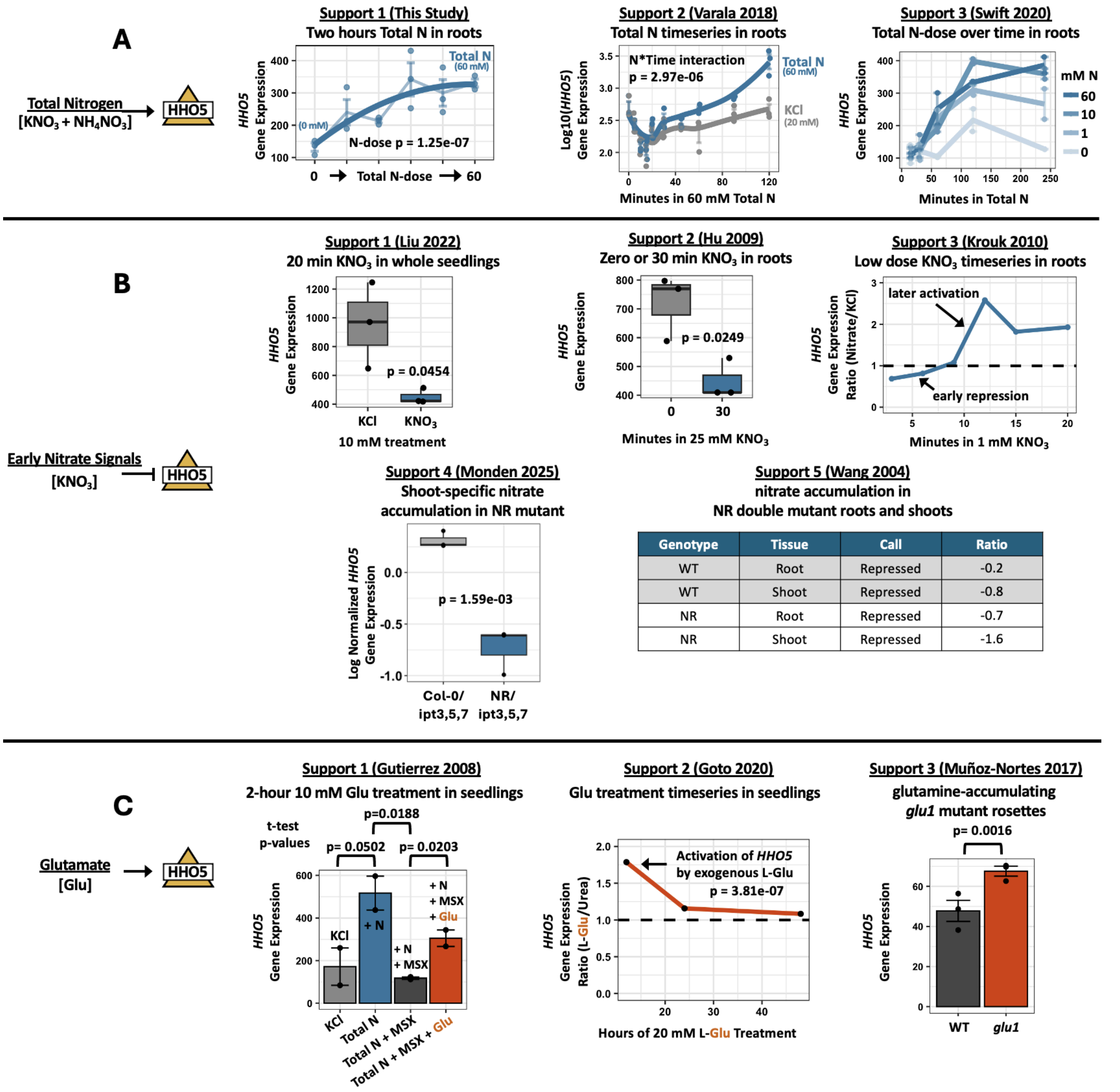

Supplementary Figure 15: *HHO5* is repressed by nitrate but induced by organic N

A) Gene expression of HHO5 in response to Total N treatments in: Dose-dependent treatments (This study), time-series treatments (Varala et al. 2018, RNA-seq), and Dose-dependent time-series treatments (Swift et al. 2020, RNA-seq). B) Gene expression of HHO5 in response to 20 minutes of 10 mM KNO3 (Lui KH et al. 2022, RNA-seq), 30 minutes of 25 mM KNO_3 (Hu et al. 2009, microarray), or 1 mM KNO_3 in a time-series (Krouk et al. 2010A, microarray). C) Expression of HHO5 in response to Total N treatments with or without MSX or glutamate (Gutierrez et al. 2008, microarray). Expression of HHO5 12, 24, and 48 hours after glutamate treatments relative to urea controls (Goto et al. 2020, RNA-seq). Gene expression in glu1 mutant rosettes (Muñoz-Nortes et al. 2017, RNA-seq). Supports Fig 8.

p-value significance of nitrate vs ammonium responses

Probe	Gene	NO3	NH4	NO3:NH4
246215_at	*HHO5*	0.5145	0.2547	0.5165
247199_at	*TGA1*	0.0012	0.0055	0.1958
264348_at	*NRT1.1*	0.0010	0.1839	0.6243

(Ristova et al 2016)

Supplementary Figure 16: *HHO5* does not respond to four hour, low dose treatments of nitrate or ammonium

*A) p-value significance or responses to nitrate, ammonium, or nitrate*ammonium interactions from the Ristova et al. 2016 microarray dataset. Supports Fig 8.*

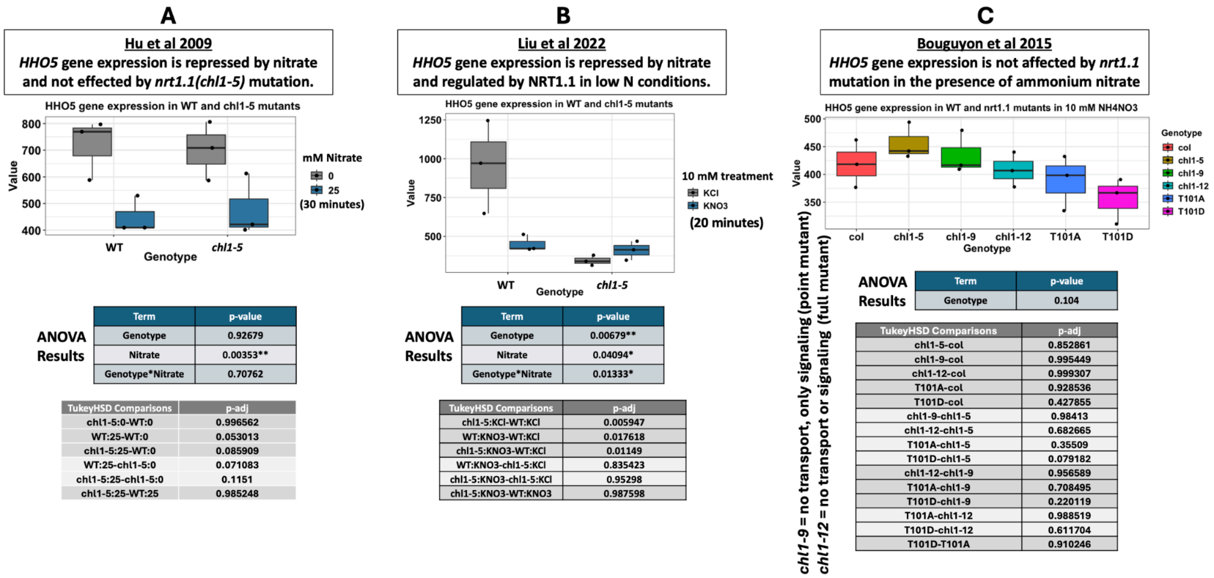

Supplementary Figure 17: *HHO5* is not consistently misregulated in *nrt1.1* mutant studies

A) HHO5 gene expression in three independent studies of nrt1.1 (chl1) mutants (Hu et al. 2009 microarray; Liu et al. 2022 RNA-seq; and Bouguyon et al. 2015 microarray). Supports Fig 8.

A

TGA1 represses *HHO5* in cells and *in planta*

Gene ID	AT5G65210 (TGA1)		AT5G65210 (TGA1)	
	Data: _ (ID: 2823)		Data: _ (ID: 2885)	
	Analysis: DESeq2_FDR<0.05		Analysis: DESeq2_FDR<0.05	
	Edges: in planta:Regulated 1 (1993)		Edges: Cells:Regulated:TARGET 1 (5749)	
	Induced-0 Repressed-1		Induced-0 Repressed-1	
**(HHO5)**	Pvalue	Log2FC	Pvalue	Log2FC
AT4G37180	6.95e-4	-9.61e-1	1.25e-5	-3.66e-1

Data source: Swift et al 2020, Image Source ConnecTF.org (Brooks et al 2021)

Michaelis-Menten modelling of *HHO5* gene expression in WT and *TGA1* perturbations *in planta*

B

MM-Modeling	Vmax	Km	Model p-value
Wild Type	1.23	0.96	0.00113
35S:TGA1	1.01	0.86	0.01466
tga1/tga4	2.39	84.25	0.02406

(Swift et al 2020)

Supplementary Figure 18: TGA1 represses the Michaelis-Menten mediated N-dose response of HHO5

*A) p-values and log2FC of HHO5 regulation by TGA1 from the ConnecTF.org database (Brooks et al. 2021). B) p-values of nitrate, ammonium, or synergistic nitrate*ammonium responses from the Ristova et al. 2016 microarray experiment. C) Michaelis-Menten kinetics of HHO5 gene expression from Swift et al. 2020. Supports Fig 8.*

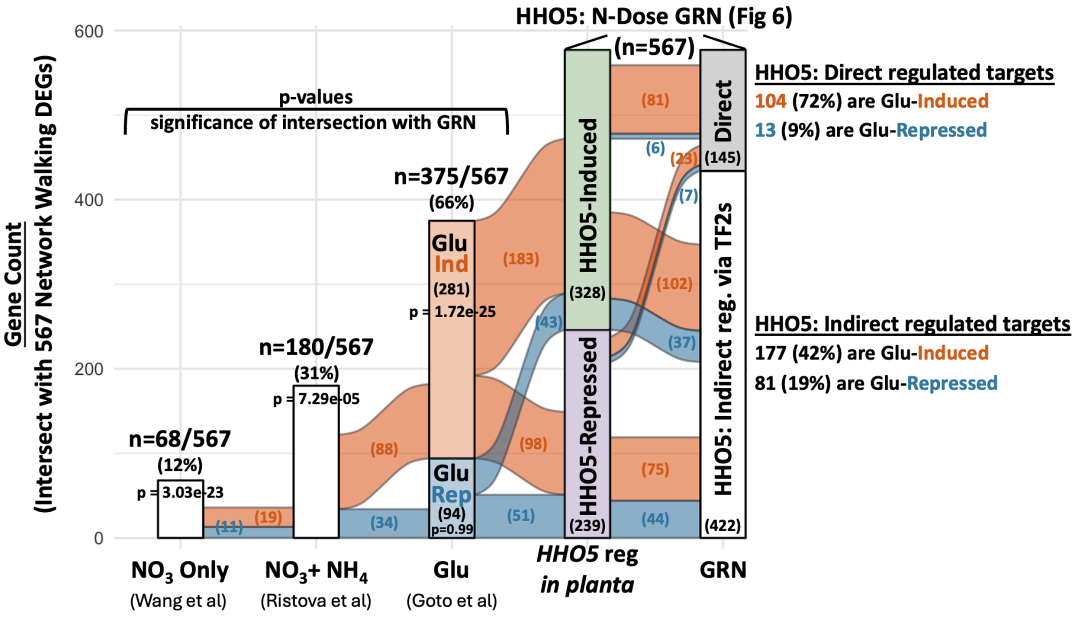

Inorganic and organic regulation of the HHO5 Network Walking GRN

Supplementary Figure 19: The HHO5 GRN is significantly enriched for nitrate-specific and glutamate induction responses

Sankey plot showing intersection of nitrate-specific genes (Wang et al. 2004), nitrate and ammonium responsive genes (Ristova et al. 2016), and glutamate response genes (Goto et al. 2020). Intersection significance represented by hypergeometric test p-values between each gene list and the 567 Network Walking N-dose genes. Ribbons represent how many genes shared between each gene list are induced or repressed by glutamate. Supports Fig 8.

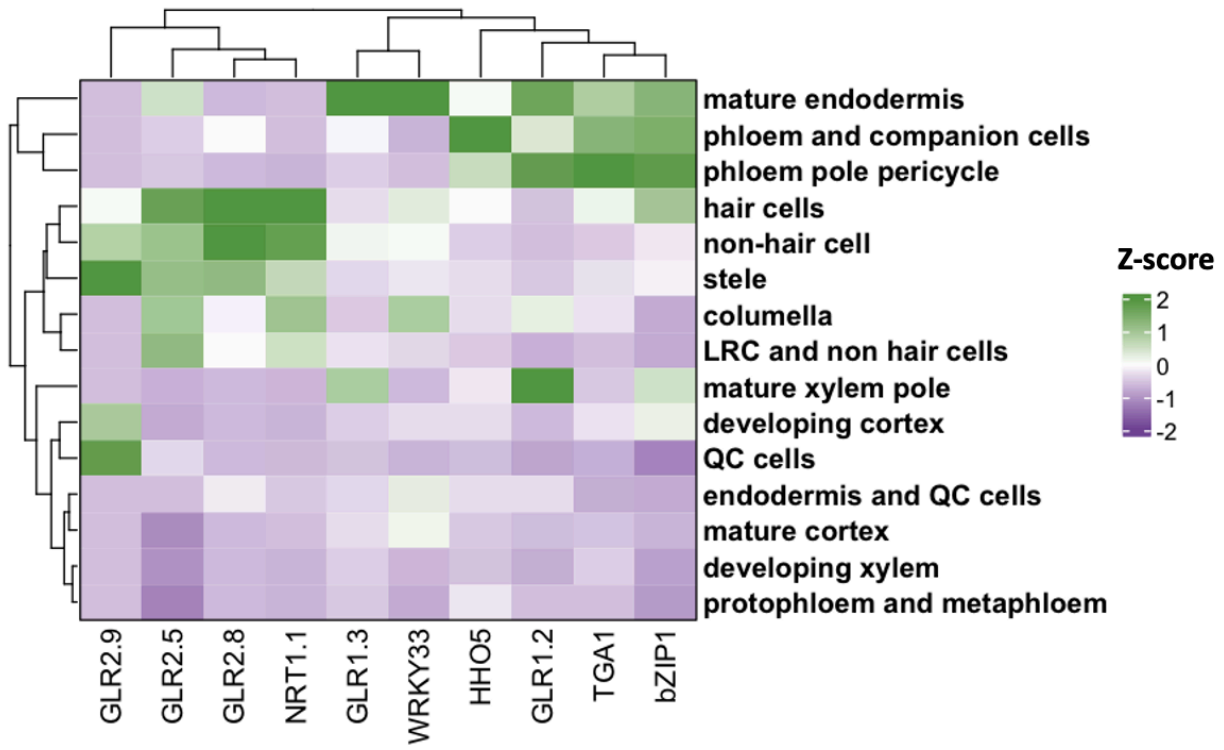

Supplementary Figure 20: *HHO5*, *bZIP1*, *TGA1*, and *GLR1.2* are expressed in phloem

A) Z-scored scaled gene expression from Li et al. 2016 of TFs of interest and the glutamate receptors (GLRs) induced by HHO5 both in cells and in planta (See Supplementary Fig. 7). Supports Fig 8.

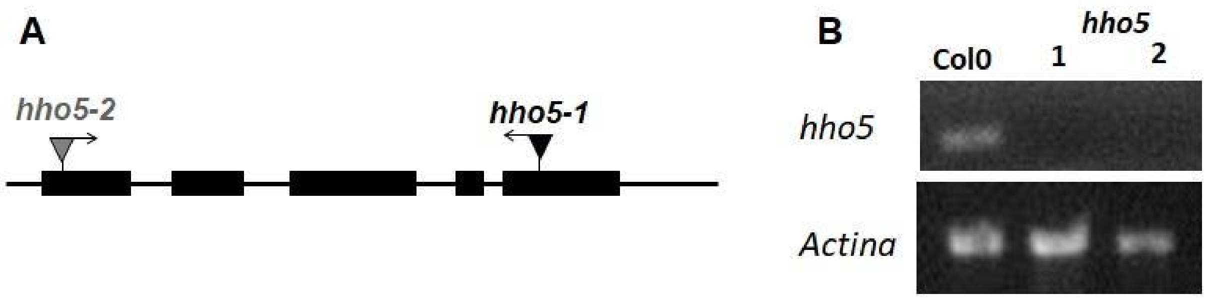

Supplementary Figure 21: Confirmation of two null HHO5 T-DNA mutants

A) The hho5-1 T-DNA is in the final exon of the gene, while the hho5-2 T-DNA is near the 5' UTR. B) RT-PCR revealed these are both null mutants, as no amplification of HHO5 was seen in either, compared to WT Col-0 plants as a control. ACTIN was used as a loading control. Supports Fig 4.
